# Integration of transcriptome and immunophenotyping data highlights differences in the pathogenetic kinetics of B cells across immune-mediated disease

**DOI:** 10.1136/rmdopen-2024-005310

**Published:** 2025-04-09

**Authors:** Shinji Izuka, Toshihiko Komai, Takahiro Itamiya, Mineto Ota, Saeko Yamada, Yasuo Nagafuchi, Hirofumi Shoda, Kosuke Matsuki, Kazuhiko Yamamoto, Tomohisa Okamura, Keishi Fujio

**Affiliations:** 1 Department of Allergy and Rheumatology, Graduate School of Medicine, The University of Tokyo, Tokyo, Japan; 2 Department of Functional Genomics and Immunological Diseases, Graduate School of Medicine, The University of Tokyo, Tokyo, Japan; 3 Research Division, Chugai Pharmaceutical Co., Ltd, Yokohama, Kanagawa, Japan; 4 Center for Integrative Medical Sciences, the Institute of Physical and Chemical Research (RIKEN), Yokohama, Kanagawa, Japan

**Keywords:** Systemic Lupus Erythematosus, Systemic Sclerosis, Rheumatoid Arthritis

## Abstract

**Objective:**

To elucidate crucial immune cell subsets and associated immunological pathways by stratifying patients with immune-mediated diseases (IMDs) using immunophenotyping and transcriptomic approaches.

**Methods:**

We conducted flow cytometric and transcriptomic analyses in 23 immune cell subsets derived from 235 patients with six IMDs, using our database, utilizing our database, ImmuNexUT. Patients were stratified based on immunophenotyping data. Subsequently, we examined clinical and transcriptomic differences among these stratified clusters.

**Results:**

Patients with IMDs were stratified into two clusters based on their immunophenotypes. Cluster 1 was enriched with differentiated B cells, including unswitched memory B cells (USM B), switched memory B cells, double-negative B cells and plasmablasts, while cluster 2 was enriched with naïve B cells. Higher disease activity in rheumatoid arthritis and decreased respiratory functions in systemic sclerosis were observed in cluster 1, whereas the disease activity of systemic lupus erythematosus was higher in cluster 2. Numerous differentially expressed genes were detected in USM B. Cluster 1 was associated with glycosylation processes in USM B and elevated B cell-activating factor signalling from myeloid cells in B cells, while cluster 2 exhibited higher B-cell receptor signalling in USM B. Patients in cluster 2, which had an elevated age-associated B-cell signature, exhibited more frequent flares, suggesting that an increased proportion of naïve B cells with this signature is associated with poor prognosis.

**Conclusion:**

Immunophenotyping-based clusters and transcriptome-based states revealed quantitative and qualitative differences in B cells. To predict IMD prognosis, assessing both the quantity and quality of naïve B cells may be crucial.

**WHAT IS ALREADY KNOWN ON THIS TOPIC**

**WHAT THIS STUDY ADDS**

- Patients with IMDs were stratified into two distinct clusters based on their immunophenotypes, revealing significant differences in B-cell subsets. Cluster 1 was enriched with mature B-cell subsets, while cluster 2 was enriched with naïve B cells.
- These clusters exhibited unique clinical features: high disease activity in rheumatoid arthritis and decreased respiratory functions in systemic sclerosis in cluster 1 versus high disease activity in systemic lupus erythematosus and higher flare rates in cluster 2.
- Cluster 1 was associated with endoplasmic reticulum stress and glycosylation pathways in unswitched memory B cells and elevated B cell-activating factor signalling from myeloid cells. In contrast, cluster 2 exhibited enhanced B-cell receptor signalling and was further stratified into subgroups based on the presence or absence of age-associated B-cell signatures within mature B cells.

**HOW THIS STUDY MIGHT AFFECT RESEARCH, PRACTICE OR POLICY**

- The study highlighted the importance of immunophenotypes of B-cell subsets across various IMDs. This approach could offer a more nuanced classification system for IMDs, supported by distinct clinical and transcriptomic features. This has the potential to advance precision medicine for the management of IMDs.

## Introduction

 Immune-mediated diseases (IMDs) are characterised by high levels of heterogeneity, with variations in pathogenesis both within and across individual diseases.^1^ Immunophenotyping of peripheral blood mononuclear cells (PBMCs) has been used to identify subgroups within various IMDs, including systemic lupus erythematosus (SLE),^2 3^ idiopathic inflammatory myopathies (IIMs),^4 5^ rheumatoid arthritis (RA),^6 7^ systemic sclerosis (SSc)^8^ and mixed connective tissue disease (MCTD).^9^ Immunophenotyping, in this context, refers to the quantification of immune cell proportions using flow cytometry, providing insights into immune cell composition. IMD patients have been stratified into immunophenotype-defined or molecularly-defined subgroups, aiming to enable precision medicine.^59^,^14^ While many studies focus on either immunophenotyping or transcriptomic analysis in single diseases or in groups with similar pathogenetic mechanisms,^1015^,^17^ comprehensive cross-disease analyses that integrate both approaches remain relatively scarce.

We recently developed the Immune Cell Gene Expression Atlas from the University of Tokyo (ImmuNexUT), which encompasses comprehensive gene expression data across a diverse range of immune cell subsets derived from PBMCs, along with whole-genome sequencing data from patients with various IMDs.^1^ Here, we conducted immunophenotyping analyses in 23 immune cell subsets across six IMDs (SLE, MCTD, IIM, SSc, RA and large vessel vasculitis (LVV)) from the ImmuNexUT cohort. These immunophenotyping-based findings, supported by transcriptomic and clinical characteristics of IMDs, may underscore the significance of B-cell subsets.

## Materials and methods

### Study design and participants

We used data from ImmuNexUT,^1^ which primarily included Japanese patients (Japanese: n=231; Chinese: n=3; Vietnamese: n=1) diagnosed with SLE, MCTD, SSc, IIM, RA and LVV, all of whom met the respective diagnostic or classification criteria.^18^,^26^ We collected prognostic data for each patient from electronic medical records. The definition of flare was as follows: for SLE, we used the Safety of Estrogens in Lupus Erythematosus, National Assessment (SELENA)-Systemic Lupus Erythematosus Disease Activity Index (SLEDAI) Flare Index, which categorises flares as mild-to-moderate or severe.^27^ For IIM, we defined flare as worsening muscle or extramuscular symptoms,^28^ requiring additional immunosuppressive therapy. In SSc, disease flare was defined as progression in one or more organ systems, including pulmonary involvement (eg, interstitial lung disease (ILD) progression or pulmonary arterial hypertension development or worsening), an increase in the modified Rodnan skin score, worsening musculoskeletal symptoms or the presence of skin ulcers.^29^ For MCTD, we used the SELENA-SLEDAI criteria^27^ along with worsening ILD requiring additional immunosuppressive therapy. In RA, flare was defined by the physician’s intention to treat, specifically the initiation or modification of biologic or targeted synthetic disease-modifying anti-rheumatic drugs.^30^ For LVV, flare was defined based on clinical features of ischaemia or evidence of active aortic inflammation, leading to progressive aortic or large-vessel dilatation, stenosis or dissection,^31^ all of which necessitate additional therapy.

**Figure 1:**
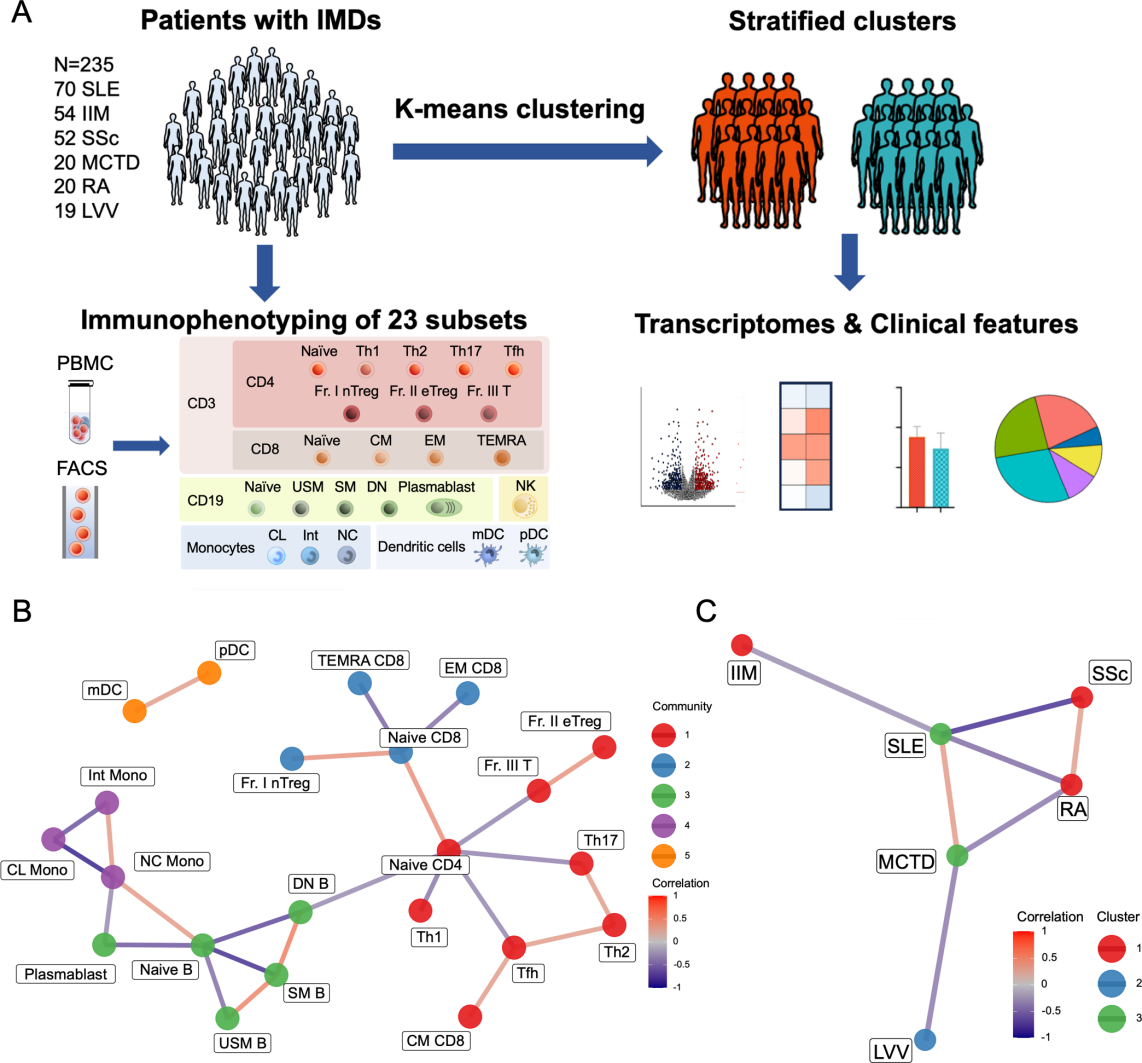
Immunophenotyping of six immune-mediated diseases. (**A**) Study workflow. A total of 235 patients diagnosed with IMDs were included, and comprehensive immunophenotyping was conducted in all patients, who were subsequently categorised into distinct clusters based on their immunophenotypes of 23 immune cell subsets. Stratified clusters were further characterised by integrating the transcriptomic data of each immune cell subset and clinical features. (**B**) Correlation network analysis of immune cell subsets based on cell proportions. The network represents Pearson correlations among the 23 immune cell subsets based on their relative proportions. The edges are coloured according to the correlation values. Nodes are grouped into community clusters identified by network analysis. Correlations with an absolute value of |r|>0.3 and adjusted p value<0.05 are shown. (**C**) Correlation network analysis of six IMDs based on the principal components of the mean cell proportion data. The edges are coloured according to the correlation values. Nodes are coloured according to their cluster membership. Correlations with an absolute value of |r|>0.25 and adjusted p value<0.05 are shown. CL, classical; CM, central; memory; DN, double-negative; EM, effector memory; IIMs, idiopathic inflammatory myopathies; IMD, immune-mediated disease; Int, intermediate; LVV, large vessel vasculitis; MCTD, mixed connective tissue disease; mDC, myeloid dendritic cells; NC, non-classical; NK, natural killer; PBMC, peripheral blood mononuclear cell; pDC, plasmacytoid dendritic cells; SM, switched memory; SSc, systemic sclerosis; TEMRA, T effector memory cell; USM, unswitched memory.

To minimise the influence of treatment, patients receiving prednisolone doses over 10 mg were excluded. Both PBMC and clinical data were also collected from these patients. We stratified all patients based on the immunophenotypes of 23 immune cell subsets. Following stratification, we performed transcriptome analyses in the 23 immune cell subsets and compared clinical features to elucidate the differences among the classified groups (figure 1A).

### Cell and plasma isolation and flow cytometry

Immediately following blood collection, PBMCs were isolated by density gradient separation using Ficoll-Paque (GE HealthCare). Erythrocytes were lysed using a potassium ammonium chloride buffer, and non-specific binding was inhibited using Fc-gamma receptor antibodies. The PBMCs were then sorted into 23 distinct immune cell subsets using a 14-colour cell sorter, the BD FACSAria Fusion (BD Biosciences). Manual gating using FlowJo Analysis Software (TreeStar, San Jose, California, USA) was performed to identify the proportions of these 23 immune cell subsets. Further details about the flow cytometry markers and gating strategy can be found in our previous study^1^ (online supplemental table 1).

The 23 immune cell subsets comprised naïve CD4^+^ T cells (naïve CD4), T helper 1, 2 and 17 cells, T follicular helper cells, fraction I naïve regulatory T cells, fraction II effector regulatory T cells, fraction III non-regulatory T cells (Fr. III T), naïve CD8^+^ T cells (naïve CD8), central memory CD8^+^ T cells, effector memory CD8^+^ T cells, CD8^+^ T effector memory CD45RA^+^ cells, natural killer cells (NK), naïve B cells (naïve B), unswitched memory B cells (USM B), switched memory B cells (SM B), double-negative B cells (DN B), plasmablasts, classical monocytes (CL Mono), intermediate monocytes (Int Mono), non-classical monocytes (NC Mono), myeloid dendritic cells (mDC) and plasmacytoid dendritic cells (pDC). The proportions of each cell subset were calculated. The definitions and parent subsets of the cell subsets are detailed in the appendix^1^ (online supplemental table 2).

The differences in cell proportions between our IMDs and other diseases were analysed using linear regression models adjusted for age, sex, dose of prednisolone and the use of immunosuppressants. Likewise, differences in cell proportions with or without specific medications were analysed using linear regression models adjusted for age, sex and disease type. We conducted network analysis among immune cells generating Pearson correlation matrices. Bonferroni correction was performed to adjust for multiple testing, and significant correlations (p<0.05) were refined to those above a predefined threshold (|r|>0.3, adjusted p<0.05) using the igraph package (V.2.0.3). Analysis of the relationships among the diseases involved several steps. First, the mean cell proportions of the 23 immune cell subsets in each disease were calculated. Next, principal component analysis (PCA) of these standardised immune cell proportions was performed to reduce dimensionality. Subsequently, Euclidean distances between the diseases were computed based on the principal components (PCs). This was followed by hierarchical clustering using Ward’s method. Finally, Pearson correlations (|r|>0.25, adjusted p<0.05) were computed to identify strong correlations between the diseases, which were then visualised in a network graph.

### Stratification of patients

We stratified all patients into two distinct clusters based on the immunophenotypes of 23 immune cell subsets using k-means clustering. The optimal number of clusters was determined using silhouette analysis in the NbClust package (V.3.0.1) and measuring the cluster stability by Jaccard similarity.^32^ Heatmaps were generated using the ComplexHeatmap (V.3.19). We additionally employed the hclust function in R to perform hierarchical clustering, using Euclidean distance to measure dissimilarity and Ward’s method for clustering. This approach was used to compare the k-means clusters and hierarchical clustering results.

### RNA sequencing

After sorting 23 immune cell subsets using flow cytometry, we performed RNA sequencing on each subset from the patient samples, obtained gene expression data for each cell type and proceeded with the analysis. Genes with low expression, defined as having fewer than 10 raw counts or fewer than 1 count per million (CPM) in more than 85% of samples, were excluded from the study as previously described.^33^ For each cell type, we performed trimmed mean of M values normalisation using the edgeR package (V.4.3). Differentially-expressed gene (DEG) analysis was also conducted using edgeR, adjusting for covariates including age, sex, prednisolone dose, use of immunosuppressants and sequencing batch. A false discovery rate threshold of 0.05 was applied using the Benjamini-Hochberg correction to determine statistical significance.^34^ The normalised expression data were log-transformed to counts per million (log (CPM+1)). Batch effects were corrected using the ComBat function from the sva package (V.3.50.0), incorporating the same covariates for DEG analysis. Subsequent analyses included Gene Ontology (GO) enrichment of biological process terms, with a q value below 0.05, via clusterProfiler (V.4.10.0) and gene set variation analysis (GSVA) using the GSVA package (V.1.50.0) across 23 immune cell subsets.

The gene sets for GSVA were selected from the Molecular Signature Database hallmark collection and included the following pathways: interleukin (IL)-2 signal transducer and activator of transcription (STAT) 5 signalling, IL-6/Janus kinase (JAK)/STAT3 signalling, interferon (IFN)-α response, IFN-γ response, mechanistic target of rapamycin complex 1 (mTORC1) signalling, oxidative phosphorylation and tumour necrosis factor-α (TNF-α) signalling via nuclear factor-kappa B.^35^ To calculate an age-associated B cell (ABC) signature, we applied previously reported genes,^36^ including *TBX21, CXCR3, ITGAX, FCRL5* and *LILRB1*, for GSVA scoring. Additionally, we defined *CD69, CD86, CD83, MIR155HG, NFKB1, FOS, JUNB* and *MYC* as B-cell activation-related genes.^37 38^

To investigate the cell–cell communication patterns among 23 immune cell types, the CellChat package (V.2.1.0) was applied. Although ligand-receptor analysis methods are commonly applied in single-cell RNA sequencing, we successfully applied this approach to sorted bulk RNA sequencing in our previous study.^33^ We first constructed a Seurat object^39^ using the bulk RNA sequencing data from 23 immune cell types. The objects were generated based on the identified sample clusters (clusters 1 and 2). For each cluster, we created a separate CellChat object. We generated heatmaps of cell–cell interaction strengths between immune cell subsets.

### Statistical analysis

We conducted statistical comparisons of clinical parameters, proportions of the 23 immune cell subsets and GSVA scores using Fisher’s exact test or the Wilcoxon test as appropriate. To maintain the robustness of our findings, we implemented Bonferroni’s correction to account for multiple comparisons when analysing the 23 immune cell proportions. This rigorous approach prioritises minimising false positives and strengthens the conclusions drawn about immunophenotype variations.^34^ Additionally, we applied z-score standardisation to cell proportions to normalise data distribution and reduce the relative impact of extreme values, while retaining all samples. Therefore, no samples were excluded as outliers, preserving the full variability of the data set. Continuous variables are expressed as medians and IQRs. Categorical variables are expressed as frequencies and percentages. We performed time-to-event analyses of flare rates by fitting Kaplan-Meier curves with the survfit function (from the survival package (V.3.8–3) and generated the corresponding plots and log-rank p values at 3 and 5 years from the disease onset.^40^ All statistical analyses were performed using R V.4.3.2, and figures were also generated using GraphPad Prism V.10.0.2.

## Results

### Immunophenotypic characteristics of the six IMDs

We analysed immunophenotyping data, along with gene expression data, for the 23 immune cell subsets derived from PBMCs. Immunophenotyping and clinical data were also collected from 235 patients with IMDs, comprising 70 patients with SLE, 54 with IIM, 52 with SSc, 20 with MCTD, 20 with RA and 19 with LVV (figure 1A, online supplemental table 3). Transcriptomic data for 23 immune cell subsets were obtained from 223 of these patients, comprising 63 patients with SLE, 54 with IIM, 52 with SSc, 18 with MCTD, 20 with RA and 16 with LVV.

The network analysis revealed unique relationships among the immune cell subsets (figure 1B). In addition to the correlations observed within the same cell subsets (T cells, B cells and myeloid cells), we found that naïve B and CL Mono were positively correlated, whereas plasmablasts and NC Mono were negatively correlated. Hierarchical clustering was performed using the first five PCs 1–5, which collectively accounted for 100% of the variance in mean cell proportions across the six IMDs. The results revealed that SLE and MCTD formed one cluster, IIM, SSc and RA formed a second cluster, and LVV formed an independent cluster (figure 1C). Furthermore, Pearson correlation analysis revealed that SLE and MCTD, as well as SSc and RA, exhibited positive correlations (figure 1C). While these correlations were based on the average cell proportions within each disease, these correlations support the clustering findings, indicating that SLE and MCTD share similar immunophenotyping, as do SSc and RA.

**Figure 2:**
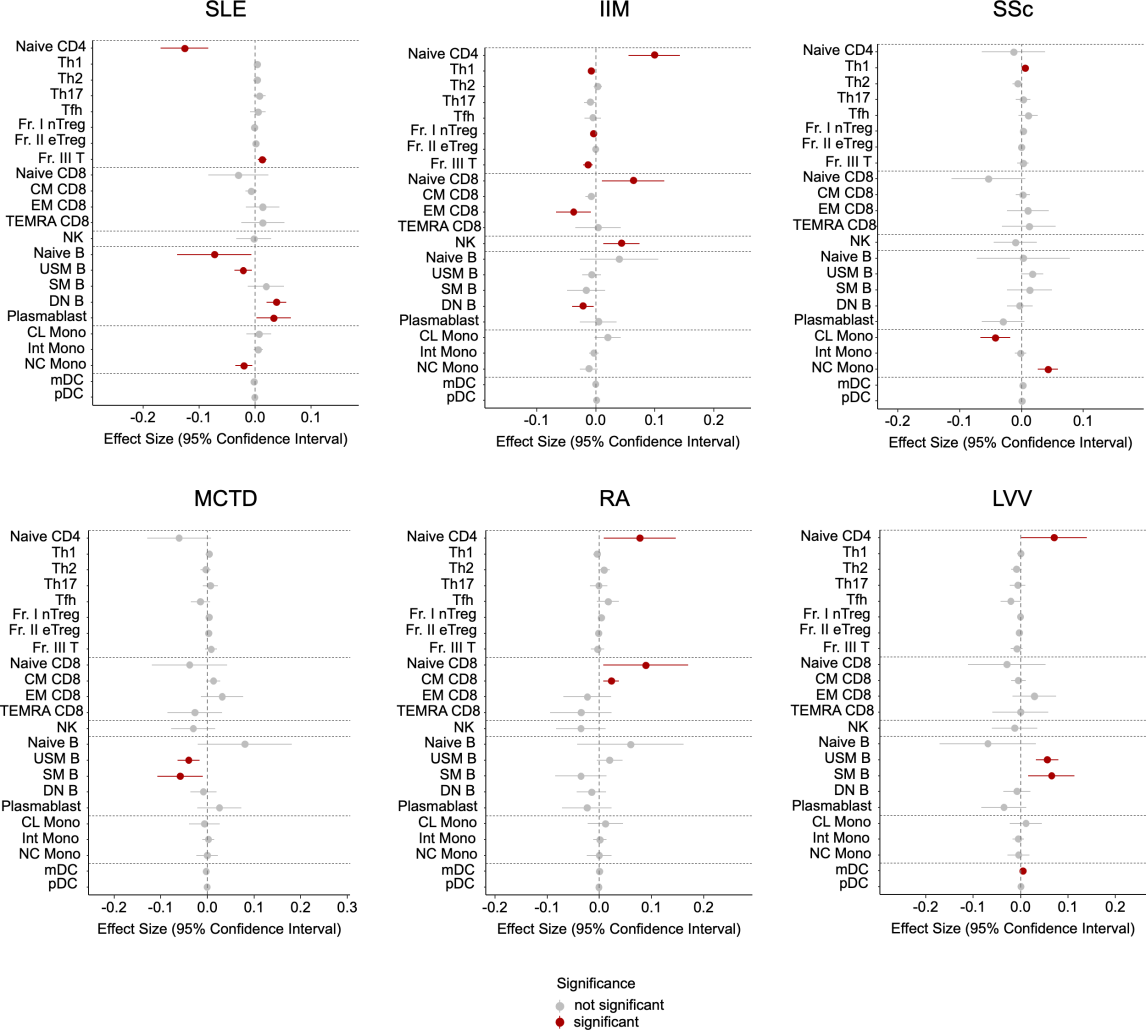
Immune cell proportions in IMDs among other diseases. Linear regression models were developed to analyse the proportions of 23 immune cell subsets in patients with six IMDs, comparing them with those of five other diseases. The predictors included in the models were age, sex, dose of prednisolone and use of immunosuppressants. CL Mono, classical monocytes; CM CD8, central memory CD8^+^ T cells; DN B, double-negative B cells; EM CD8, effector memory CD8^+^ T cells; Fr. I nTreg, fraction I naïve regulatory T cells; Fr. II eTreg, fraction II effector regulatory T cells; Fr. III T, fraction III non-regulatory T cells; IIM, idiopathic inflammatory myopathy; IMD, immune-mediated disease; Int Mono, intermediate monocytes; LVV, large vessel vasculitis; MCTD, mixed connective tissue disease; mDC, myeloid dendritic cells; naïve B, naïve B cells; naïve CD4, naïve CD4^+^ T cells; naïve CD8, naïve CD8^+^ T cells; NC Mono, non-classical monocytes; NK, natural killer; PC, principal component; pDC, plasmacytoid dendritic cells; RA, rheumatoid arthritis; SLE, systemic lupus erythematosus; SM B, switched memory B cells; SSc, systemic sclerosis; TEMRA CD8, CD8^+^ T effector memory CD45RA^+^ cells; Tfh, T follicular helper cells; Th1, T helper 1 cells; Th2, T helper 2 cells; Th17, T helper 17 cells; USM B, unswitched memory B cells.

Next, we identified unique immune cell subsets in each IMD using linear regression models adjusted for age, sex, prednisolone dose and immunosuppressant use, with comparisons among the six diseases (figure 2). As each patient exhibits a heterogeneous immunophenotyping pattern, this analysis highlights distinct immunological differences in specific cell subsets between diseases. Unlike the overall similarity observed among diseases at the group level (figure 1C), this analysis provides a more granular view of disease-specific immune profiles. For example, patients with SLE predominantly exhibited increased proportions of Fr. III T, DN B and plasmablasts and decreased proportions of naïve B, USM B and NC Mono. Patients with IIM had higher proportions of naïve CD4, naïve CD8 and NK. Patients with SSc showed a high proportion of NC Mono, while MCTD was characterised by decreased USM B and SM B proportions. Similar to IIM, RA was associated with increased naïve CD4 and naïve CD8 proportions. Lastly, patients with LVV exhibited significantly increased proportions of USM B and SM B.

We further analysed the estimated effects of medications on immunophenotyping (online supplemental figure 1). Notable findings were observed, particularly in B-cell populations. Prednisolone was associated with a significant reduction in naïve B cells, while mycophenolate mofetil and methotrexate were linked to decreased plasmablast proportions. Additionally, azathioprine and calcineurin inhibitors were associated with a reduction in naïve B and an increase in SM B.

Although each disease possessed unique characteristics, a closer examination of the individual patients revealed distinct immunophenotypic profiles even within the same disease and shared immunophenotypic profiles across different diseases (online supplemental figure 2). This underscores the inherent heterogeneity of IMDs and supports the stratification of patients based on immunophenotype.

### Stratifying patients with IMDs based on immunophenotypes

**Figure 3:**
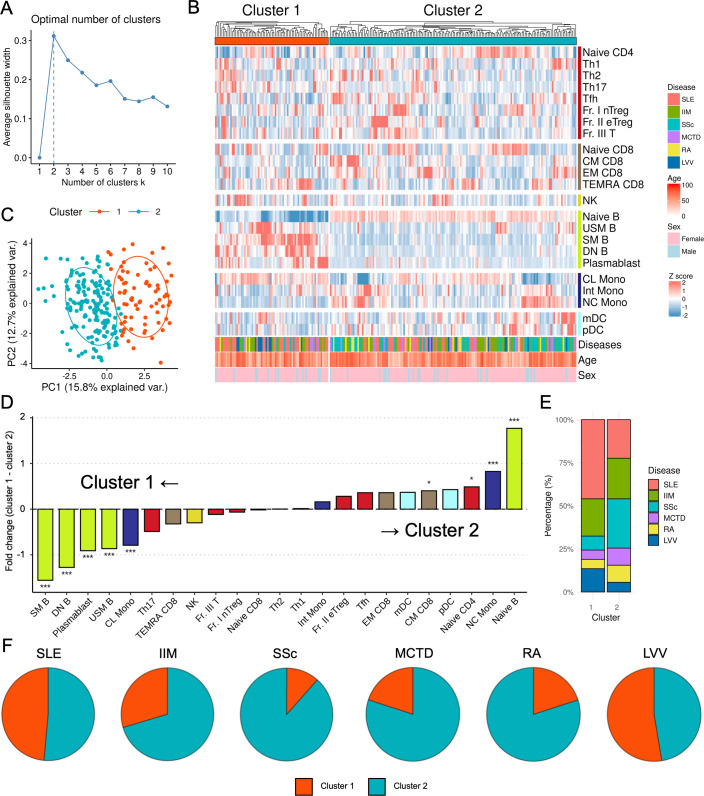
Stratification of immune-mediated diseases according to immunophenotype. (**A**) Silhouette plot of the average silhouette width for k-means clustering with different numbers of clusters. The highest silhouette value of 2 indicates that two clusters provide the best balance between within-cluster cohesion and between-cluster separation. (**B**) Heatmap of immune cell proportions across clusters. The scaled z-scores of 23 immune cell subsets across the two clusters were identified by k-means clustering. (**C**) PCA of the immunophenotypic profiles of the IMD patients. Patients are colour-coded by their cluster membership. (**D**) The fold change in mean cell proportions across the 23 immune cell subsets between the two patient clusters. Comparisons between clusters for each cell subset were conducted using the Wilcoxon test, adjusted using Bonferroni’s correction (*p<0.05, ***p<0.001). (**E**) Disease composition within the two clusters. (**F**) Pie charts showing the proportion of patients with each disease in the two clusters, displaying the proportion of different IMDs in two clusters identified by k-means clustering. CL Mono, classical monocytes; CM CD8, central memory CD8^+^ T cells; DN B, double-negative B cells; EM CD8, effector memory CD8^+^ T cells; Fr. I nTreg, fraction I naïve regulatory T cells; Fr. II eTreg, fraction II effector regulatory T cells; Fr. III T, fraction III non-regulatory T cells; IIM, idiopathic inflammatory myopathy; IMD, immune-mediated disease; Int Mono, intermediate monocytes; LVV, large vessel vasculitis; MCTD, mixed connective tissue disease; mDC, myeloid dendritic cells; naïve B, naïve B cells; naïve CD4, naïve CD4^+^ T cells; naïve CD8, naïve CD8^+^ T cells; NC Mono, non-classical monocytes; NK, natural killer; PC, principal component; PCA, principal component analysis; pDC, plasmacytoid dendritic cells; RA, rheumatoid arthritis; SLE, systemic lupus erythematosus; SM B, switched memory B cells; SSc, systemic sclerosis; TEMRA CD8, CD8^+^ T effector memory CD45RA^+^ cells; Tfh, T follicular helper cells; Th1, T helper 1 cells; Th2, T helper 2 cells; Th17, T helper 17 cells; USM B, unswitched memory B cells.

To reclassify all the patients based on more closely-related pathogeneses, we conducted clustering analysis using immunophenotyping data. First, silhouette analysis was performed to determine the optimal number of clusters, which was two (figure 3A). In addition, two or three clusters exhibited higher Jaccard stability (online supplemental figure 3). Therefore, we applied k-means clustering to categorise all 235 patients into two distinct clusters (figure 3B). PCA revealed well-demarcated regions corresponding to each cluster (figure 3C). PC1 displayed a descending order of absolute values for naive B, SM B, DN B, NC Mono, CL Mono, USM B and plasmablasts (online supplemental table 4). Variations in immune cell subset distributions were also apparent, with the B-cell subsets included in PC1 showing particularly distinct segregation of the two clusters (figure 3D, online supplemental table 5). Specifically, cluster 1 was enriched with SM B, DN B, plasmablasts and USM B, whereas cluster 2 was characterised by a predominance of naïve B and NC Mono. These findings underscore the potential role of B-cell subsets in distinguishing patients with IMDs.

Using non-hierarchical clustering based on immunophenotyping, we identified two clusters comprising 66 patients in cluster 1 and 169 patients in cluster 2 (figure 3E, table 1). To validate our clustering approach, we also performed hierarchical clustering and observed a high match rate (81.3%) with k-means clustering (online supplemental figure 4). Distinct disease-specific patterns were observed between the patients of each cluster. Cluster 1 had a significantly higher proportion of patients with SLE compared with other diseases (45.2%, p=0.004) (figure 3E). SLE and LVV patients were nearly evenly distributed between the two clusters (figure 3F, table 1). In contrast, the majority of patients with SSc were in cluster 2 (88.5%, p=0.003).

**Table 1:** Clinical characteristics and disease distribution of the two clusters

	Cluster 1 (n=66)	Cluster 2 (n=169)	P value
**Male sex (%**)	7 (10.6)	26 (15.4)	0.408
**Age, median (IQR) years**	56.00 (44.00–70.00)	57.00 (45.00–69.00)	0.862
**Disease duration, median (IQR) years**	17.00 (7.25–23.75)	11.00 (2.00–20.00)	0.013
**Disease (%**)			0.002
**SLE (%**)	28 (42.4)	42 (24.9)	0.0037
**IIM (%**)	14 (21.2)	40 (23.7)	0.733
**SSc (%**)	6 (9.1)	46 (27.2)	0.0026
**MCTD (%**)	4 (6.1)	16 (9.5)	0.603
**RA (%**)	4 (6.1)	16 (9.5)	0.603
**LVV (%**)	10 (15.2)	9 (5.3)	0.03
**CRP median (IQR**)	0.07 (0.04–0.18)	0.11 (0.04–0.47)	0.071
**ESR median (IQR) mm/hr**	16.00 (8.50–34.00)	21.50 (12.25–45.00)	0.044
**IgG median (IQR) mg/dL**	1225.50 (938.25–1515.00)	1464.50 (1211.00–1817.75)	0.001
**Treatment**			
**Dose of PSL, median, (IQR) mg**	5.00 (4.81–7.00)	0.00 (0.00–5.00)	<0.001
**Hydroxychloroquine (%**)	10 (15.2)	16 (9.5)	0.248
**Tacrolimus (%**)	12 (18.2)	17 (10.1)	0.121
**Cyclosporine A (%**)	8 (12.1)	6 (3.6)	0.026
**Mycophenolate mofetil (%**)	4 (6.1)	9 (5.3)	0.761
**Azathioprine (%**)	15 (22.7)	2 (1.2)	<0.001
**Methotrexate (%**)	10 (15.2)	19 (11.2)	0.508

CRP, C-reactive protein; ESR, erythrocyte sedimentation rate; IIM, idiopathic inflammatory myopathy; LVV, large vessel vasculitis; MCTD, mixed connective tissue disease; PSL, prednisolone; RA, rheumatoid arthritis; SLE, systemic lupus erythematosus; SSc, systemic sclerosis.

### Clinical relevance of the immunophenotyping-based clusters

**Figure 4:**
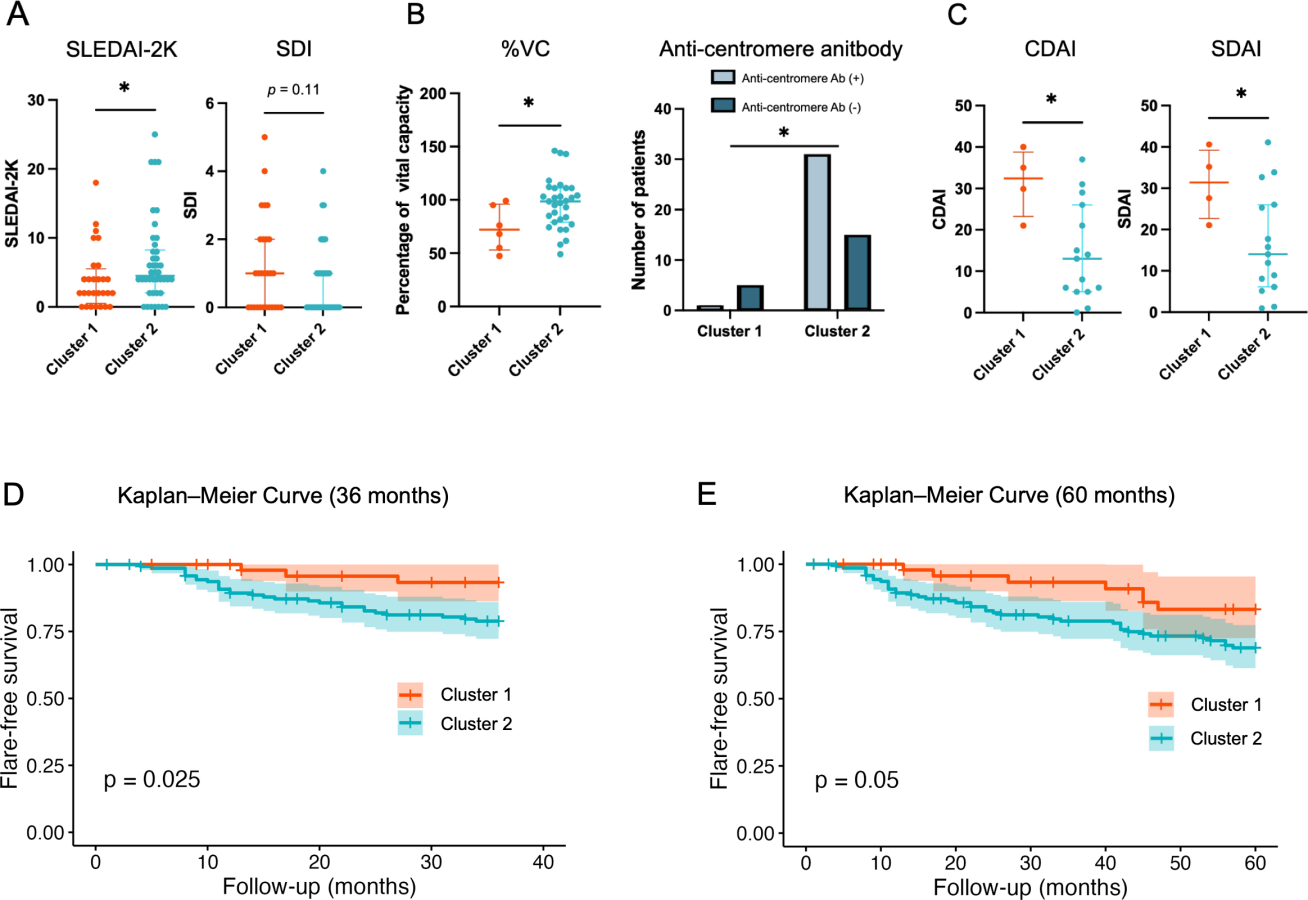
Comparison of clinical and serological characteristics and flare rates between the two clusters. (**A**) Clinical differences in SLE, showing the SLEDAI and SDI scores. (**B**) Clinical differences in SSc, showing the %VC in each cluster and the proportion of patients with or without anti-centromere antibodies. (**C**) Clinical differences in RA, showing the CDAI and SDAI scores. (*p<0.05). (**D**) At 36 months and (**E**) at 60 months, Kaplan-Meier curves comparing flare-free survival between cluster 1 and cluster 2. CDAI, Clinical Disease Activity Index; SDAI, Simplified Disease Activity Index; SDI, Systemic Lupus International Collaborating Clinics (SLICC) Damage Index; SLEDAI, Systemic Lupus Erythematosus Disease Activity Index; %VC, per cent vital capacity.

To confirm whether this immunophenotyping-based classification is clinically meaningful, we assessed the differences in the clinical characteristics of the clusters (figure 4, online supplemental table 6–11).

### Systemic lupus erythematosus

Patients with SLE in cluster 1 had a higher Systemic Lupus International Collaborating Clinics Damage Index (SDI) score compared with patients in cluster 2 (1.00 (0.00, 2.00) vs 0.00 (0.00, 1.00), p=0.106) (figure 4A, online supplemental table 6). These patients also exhibited higher titres and prevalence of anti-SS-A antibodies (66.10 IU/mL (12.30, 240.00) vs 26.05 IU/mL (1.37, 240.00), p=0.192, 78.9% vs 52.8%, p=0.082), though these differences did not reach statistical significance (online supplemental table 6). In contrast, patients with SLE in cluster 2 exhibited significantly higher disease activity, as reflected by an elevated SEDAI-2000 (SLEDAI-2K) score (4.50 (2.50, 8.00) vs 2.00 (1.50, 4.50), p=0.042) and higher Physician Global Assessment scores (3.00 (1.00, 6.00) vs 1.00 (0.00, 3.00), p=0.006) (figure 4A, online supplemental table 6). Serologically, cluster 2 was characterised by a higher prevalence of anti-dsDNA antibodies (42.9% vs 25.0%, p=0.203) and significantly higher positivity for anti-cardiolipin antibodies (53.1% vs 19.0%, p=0.021) along with elevated anti-cardiolipin antibody levels (10.00 U/mL (8.00, 24.75) vs 8.00 U/mL (8.00, 8.00), p=0.007) (online supplemental table 6). Furthermore, a trend towards increased positivity for anti-β2-glycoprotein-I antibodies was observed in cluster 2 (28.9% vs 8.0%, p=0.059), which may indicate a stronger association with antiphospholipid syndrome-related features (online supplemental table 6). These findings suggest that cluster 2 represents an immunologically distinct subgroup with higher disease activity and a potential predisposition for antiphospholipid-related complications, whereas cluster 1 appears to reflect a phenotype with greater cumulative damage with anti-SS-A antibody but lower current disease activity.

### Idiopathic inflammatory myopathies

Although no statistically significant difference was observed among patients with IIM, the proportion of patients sampled at disease onset was higher in cluster 2 (50% vs 21.4%, p=0.115) (online supplemental table 7). Additionally, both current and peak creatine kinase levels were higher in cluster 2 (89.50 U/L (62.75, 144.50) vs 130.00 U/L (74.00, 888.00), p=0.118; and 315.00 U/L (92.00, 1689.00) vs 1094.50 U/L (233.75, 2601.50), p=0.172). These findings suggest that patients with IIM in cluster 2 may have experienced more severe muscle damage at the time of sampling or disease onset.

### Systemic sclerosis

The majority of patients with SSc were classified in cluster 2 (88.5%, n=46/52, p=0.003). While patients with SSc in cluster 1 showed a higher positivity rate for anti-Scl-70 and anti-RNA polymerase III antibodies, patients in cluster 1 had a significantly higher prevalence of anti-centromere antibodies compared (67.4% vs 16.7%, p=0.026) (figure 4B, online supplemental table 8). A higher proportion of patients in cluster 1 developed shortness of breath (66.7% vs 34.8%, p=0.19) and ILD (83.3% vs 39.1%, p=0.11). Moreover, these patients exhibited significantly lower per cent vital capacity (%VC) (72.00% (58.25, 90.25) vs 98.50% (80.30, 111.00), p=0.027), supporting a more severe ILD phenotype in cluster 1. Similarly, Krebs von den Lungen-6 levels, a marker of lung fibrosis, were higher in cluster 1 (497.50 U/mL (383.75, 560.25) vs 319.00 U/mL (207.00, 501.00), p=0.339), though the difference was not statistically significant (online supplemental table 8).

### Rheumatoid arthritis

Similar to SSc patients, patients with RA in cluster 1, compared with cluster 2, demonstrated significantly higher disease activity, as indicated by the higher Clinical Disease Activity Index (CDAI) score (27.67 (2.45, 36.25) vs 13.00 (5.50, 23.50), p=0.020) and Simplified Disease Activity Index (SDAI) score (31.39 (25.97, 36.53) vs 14.00 (7.12, 25.66), p=0.049) (figure 4C, online supplemental table 10). Although the difference was not statistically significant, there was a trend towards higher C-reactive protein and erythrocyte sedimentation rate levels in the patients with RA in cluster 2, which might suggest higher systemic inflammation in this group (online supplemental table 10) (p=0.147 and p=0.19, respectively). The frequencies of anti-cyclic citrullinated peptide (CCP) antibodies and rheumatoid factor were comparable across the clusters.

### Mixed connective tissue disease

In our previous study reclassifying patients with MCTD, we stratified them into SLE-like, IIM-like and SSc-like immunophenotypic subgroups.^9^ Notably, all IIM-like (2/2, 100%) and SSc-like (3/3, 100%) MCTD patients were assigned to cluster 2, which predominantly comprised IIM and SSc cases. In contrast, SLE-like MCTD patients were distributed across both clusters, with 4 out of 15 (26.7%) in cluster 1 and the remaining 11 (73.3%) in cluster 2, indicating greater heterogeneity within the SLE-like subgroup.

### Flares dynamics in each cluster

The mean follow‐up duration was 56.8 months for cluster 1 and 53.7 months for cluster 2. To investigate flare dynamics in these clusters, we performed time‐to‐event analyses using Kaplan-Meier survival curves over two distinct follow‐up periods: 36 months and 60 months. At both the 36‐month and 60‐month time points, the Kaplan-Meier curves revealed a statistically significant difference in flare‐free survival between cluster 1 and cluster 2 (log‐rank test, p=0.025 at 36 months and p=0.05 at 60 months) (figure 4D and E, online supplemental figure 5, online supplemental table 1^2^). These findings suggest that patients in cluster 2 had an overall higher risk of experiencing flares during the follow‐up period, which was evident at both the mid‐term (36‐month) and longer‐term (60‐month) assessments.

### Transcriptomic features of each disease according to cluster

**Figure 5:**
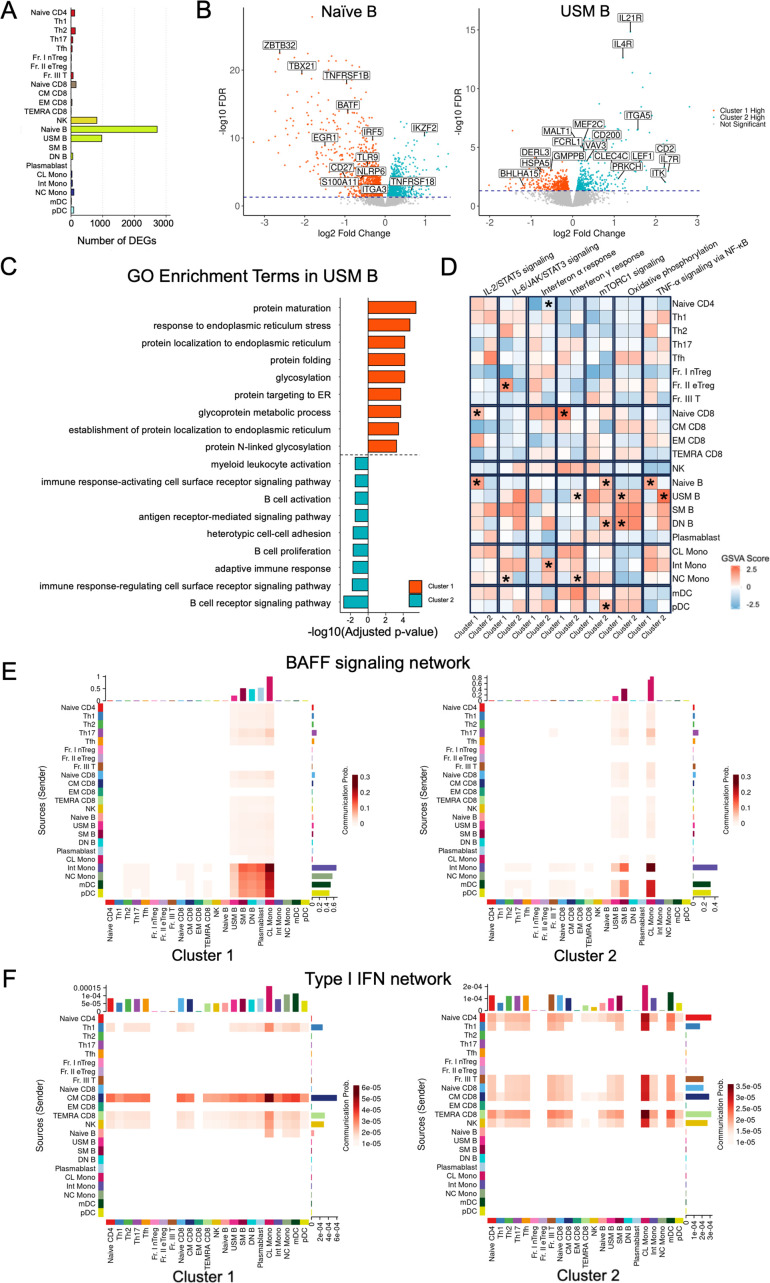
Transcriptomic characteristics of the two clusters. (**A**) Number of DEGs in the 23 immune cell subsets. (**B**) Volcano plots of DEGs between clusters 1 and 2 in naïve B and USM B. (**C**) Gene Ontology enrichment analysis of positive DEGs in cluster 1 and cluster 2 in USM B. (**D**) Heatmap of the median GSVA scores of the two clusters, comparing the key signalling pathways across the immune cell subsets between the two distinct patient clusters. Significantly different pathways are marked with an asterisk (*p<0.05). (**E**) BAFF signalling and (**F**) Type I IFN network analysis in clusters 1 and 2. Heatmaps represent the strength and direction of communication among different immune cell types. BAFF, B-cell activating factor; CL Mono, classical monocytes; CM CD8, central memory CD8^+^ T cells; DEG, differentially expressed gene; DN B, double-negative B cells; EM CD8, effector memory CD8^+^ T cells; Fr. I nTreg, fraction I naïve regulatory T cells; Fr. II eTreg, fraction II effector regulatory T cells; Fr. III T, fraction III non-regulatory T cells; GSVA, gene set variation analysis; IIM, idiopathic inflammatory myopathy; IL-2/STAT5, interleukin-2 signal transducer and activator of transcription 5; IL-6/JAK/STAT, interleukin-6/Janus kinase/signal transducer and activator of transcription 3; IMD, immune-mediated disease; Int Mono, intermediate monocytes; mDC, myeloid dendritic cells; mTORC1, mechanistic target of rapamycin complex 1; naïve B, naïve B cells; naïve CD4, naïve CD4^+^ T cells; naïve CD8, naïve CD8^+^ T cells; NC Mono, non-classical monocytes; NF-κB, nuclear factor-kappa B; NK, natural killer; PC, principal component; PCA, principal component analysis; pDC, plasmacytoid dendritic cells; RA, rheumatoid arthritis; SLE, systemic lupus erythematosus; SM B, switched memory B cells; SSc, systemic sclerosis; TEMRA CD8, CD8^+^ T effector memory CD45RA^+^ cells; Tfh, T follicular helper cells; Th1, T helper 1 cells; Th2, T helper 2 cells; Th17, T helper 17 cells; TNF-α, tumour necrosis factor-α; USM B, unswitched memory B cells.

To explore the pathogenetic mechanisms unique to each cluster, we conducted transcriptomic analyses to determine gene expression profiles in the 23 immune cell subsets. The number of DEGs varied between the clusters (figure 5A). Numerous DEGs were identified in naïve B and USM B, mirroring the variations in cell proportions observed between the two clusters. Furthermore, a pronounced difference in DEGs was detected in the naïve B of cluster 1 and USM B of cluster 2 (figure 5B). While naïve B of cluster 1 did not exhibit any significant enrichment of GO terms among the positively regulated DEGs, naïve B of cluster 2 showed an upregulation of genes related to cytokine production and cell–cell adhesion (online supplemental figure 6). In particular, USM B cells demonstrated distinct transcriptomic signatures (figure 5B). GO enrichment analysis revealed that cluster 1 was enriched in genes related to protein maturation, response to endoplasmic reticulum stress, and glycosylation, whereas cluster 2 was enriched in genes associated with B-cell receptor signalling and myeloid leucocyte activation (figure 5C).

GSVA, an unsupervised method to assess pathway enrichment across individual samples, further delineated distinct transcriptomic profiles within each cluster (figure 5D). Cluster 1 was characterised by enhanced oxidative phosphorylation in USM B and DN B, TNF-α signalling in naïve B and IFN-γ response in naïve CD8. Additionally, IL-2/STAT5 signalling was upregulated in both naïve CD8 and naïve B cells within this cluster. Conversely, the GSVA scores of cluster 2 indicated increased mTORC1 signalling in naïve B and DN B and increased TNF-α signalling in USM B. Furthermore, naïve CD4 and Int Mono in cluster 2 showed an upregulated IFN-α response, while USM B and NC Mono exhibited a pronounced IFN-γ response (figure 5D).

To further investigate interactions between B-cell subsets and other immune cells, we performed cell–cell communication inference analysis using the CellChat package, a tool designed to analyse intercellular communication networks based on ligand-receptor interactions. In cluster 1, higher levels of B cell-activating factor (BAFF) signalling were observed in NC Mono, Int Mono, mDC and pDC, with DN B cells and plasmablasts serving as potential recipients of the signalling, a phenomenon that was not confirmed in cluster 2 (figure 5E). In contrast, a wide variety of CD4^+^ T, CD8^+^ T and NK cells influenced other immune cell subsets by intercellular crosstalk via type I IFN signalling (figure 5F), suggesting different cell–cell interactions between the clusters.

**Figure 6:**
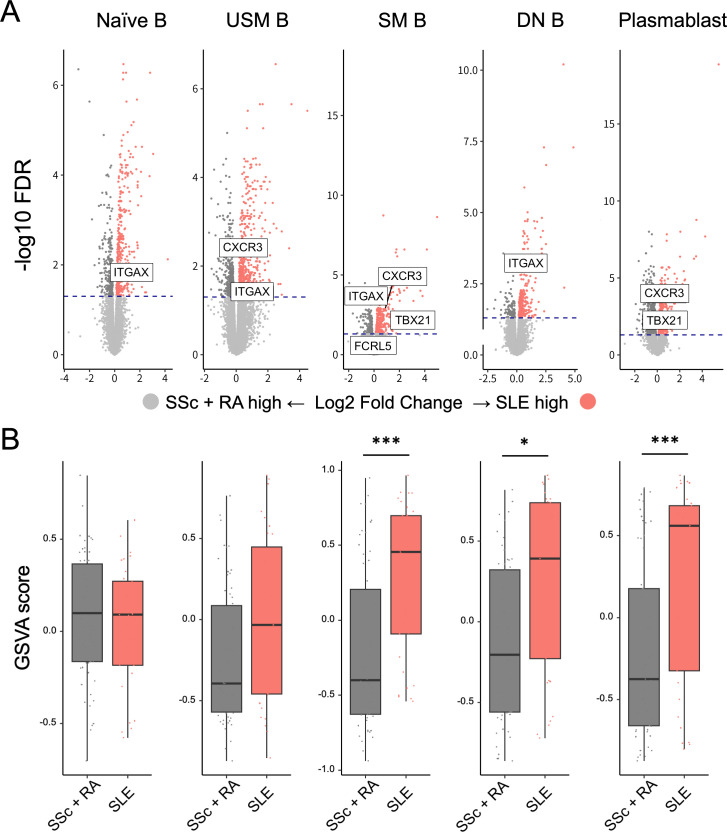
Age-associated B-cell signatures in cluster 2. (**A**) DEGs between SLE versus SSc+RA in the B-cell subsets in cluster 2. ABC-related genes, including *ITGAX, CXCR3, TBX21* and *FCRL5*, are highlighted. (**B**) GSVA scores of ABC signatures in SLE versus SSc+RA across the B-cell subsets. The ABC signature was calculated using *TBX21, CXCR3, ITGAX, FCRL5* and *LILRB1* (***p<0.001, *p<0.05). ABC, age-associated B cell; DEG, differentially expressed gene; DN B, double-negative B cells; GSVA, gene set variation analysis; naïve B, naïve B cells, RA, rheumatoid arthritis; SLE, systemic lupus erythematosus; SM B, switched memory B cells; USM B, unswitched memory B cells.

As ABC have been increasingly recognised for their pathogenic roles in IMDs,^41^,^43^ we examined the ABC signature across B-cell subsets in this cohort, as ABCs were not specifically sorted. Notably, genes such as *TBX21*, *ITGAX*, *CXCR3*, *LILRB1* and *FCRL5* were upregulated DEGs within cluster 2 compared with cluster 1 (online supplemental figure 7), suggesting an enrichment of ABC-related signatures in cluster 2. However, in cluster 2, disease activity was heightened in SLE but decreased in RA and lung function was preferable in SSc (figure 4). To further disentangle these differences, we performed a transcriptome-wide analysis comparing SLE versus RA and SSc. Interestingly, SLE had significantly elevated gene expression profiles associated with ABC across B-cell subsets^36^ (figure 6A). Moreover, in cluster 2, GSVA also revealed a marked increase in ABC-related signatures in SM B, DN B and plasmablasts in SLE compared with SSc and RA (figure 6B). In cluster 2, which included mild RA and SSc, the overall ABC-related signature was higher compared with cluster 1. However, within this cluster, patients with RA and SSc—who had lower disease activity—did not exhibit enriched ABC-related signatures in their B-cell subsets. Consistent with the enriched ABC signature, increased expression of B-cell activation genes such as *CD69* and *CD86* was observed in naïve B, USM B, SM B and DN B in cluster 2, despite a reduced proportion of USM B, SM B, DN B and plasmablasts in this cluster (online supplemental figure 8). Among SLE cases, ABC signature scores calculated using GSVA showed no significant correlation or association with SLEDAI or SDI in B-cell subsets (online supplemental table 13).

**Figure 7:**
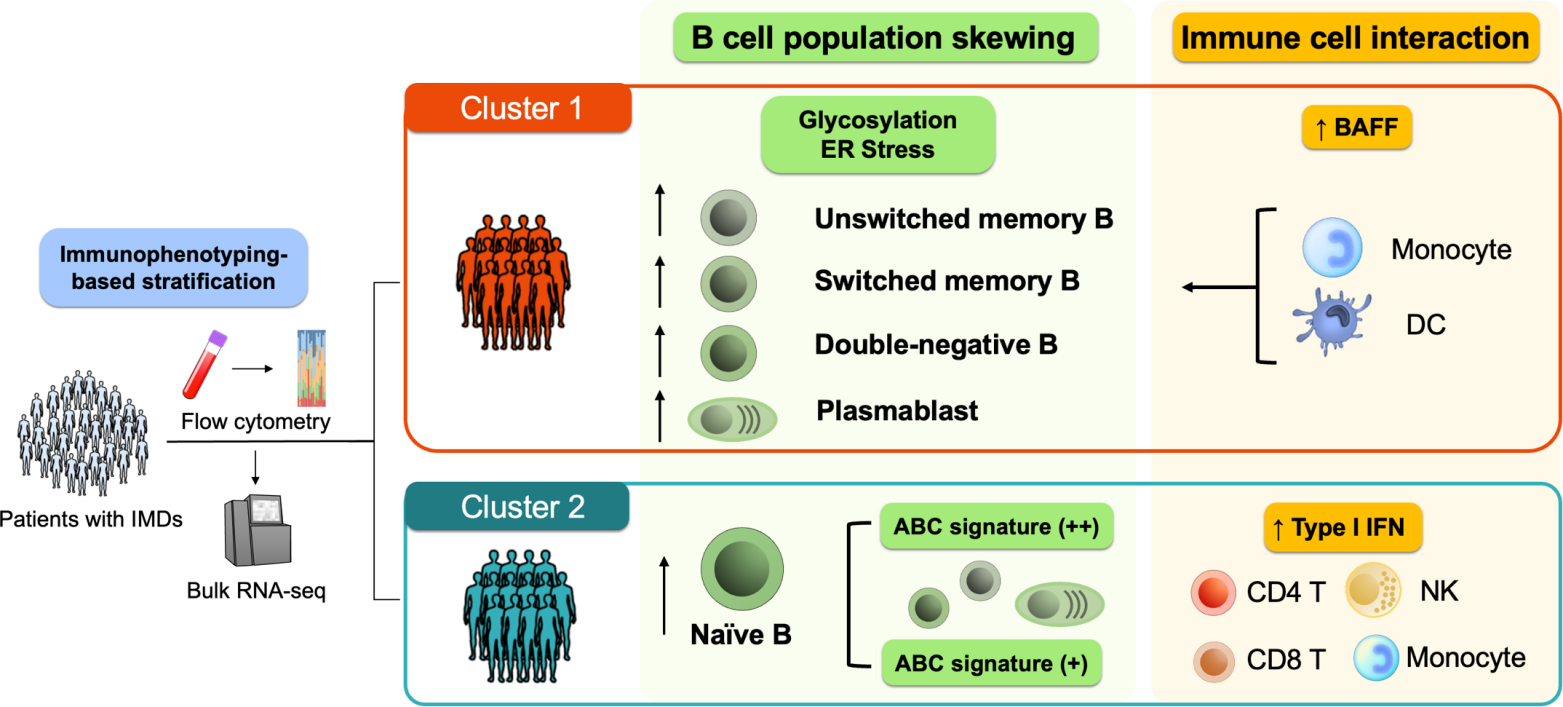
Graphical summary of the present study. We performed immunophenotyping-based stratification of patients with IMDs into two distinct clusters. Patients were stratified into a cluster enriched in differentiated B-cell subsets (cluster 1) and a cluster composed of a high proportion of naïve B cells (cluster 2). USM B in cluster 1 were enriched in glycosylation and ER stress-related pathways, with prominent BAFF signalling from myeloid cells. Cluster 2 was enriched in naïve B with higher type I IFN signalling from various immune cells. ABC, age-associated B cell; BAFF, B cell-activating factor; CL Mono, classical monocytes; CM CD8, central memory CD8^+^ T cells; DC, dendritic cell; DN B, double-negative B cells; EM CD8, effector memory CD8^+^ T cells; ER, endoplasmic reticulum; Fr. I nTreg, fraction I naïve regulatory T cells; Fr. II eTreg, fraction II effector regulatory T cells; Fr. III T, fraction III non-regulatory T cells; IFN, interferon; IMD, immune-mediated disease; Int Mono, intermediate monocytes; mDC, myeloid dendritic cells; naïve B, naïve B cells; naïve CD4, naïve CD4^+^ T cells; NC Mono, non-classical monocyte; NK, natural killer cell; pDC, plasmacytoid dendritic cells; Plasmablasts, plasmablasts; RNA-seq, RNA sequencing; SM B, switched memory B cell; Th1, T helper 1 cells; Th2, T helper 2 cells; Th17, T helper 17 cells; Tfh, T follicular helper cells; TEMRA CD8, CD8^+^ T effector memory CD45RA^+^ cells; USM B, unswitched memory B cells.

Collectively, these data suggest distinct activation states and functional orientations of immune cells between the clusters, highlighting potential mechanistic pathways that could serve as clues for future therapeutic targets (figure 7).

## Discussion

Our study delineated the pathophysiological diversity of several IMDs by analysing immunophenotyping and transcriptomic data, highlighting the critical role of B-cell subsets in IMD pathology and uncovering key transcriptomic differences relevant to disease mechanisms. These findings offer potential implications for more targeted therapeutic approaches. Additionally, the emerging success of chimeric antigen receptor T-cell therapy targeting B cells in treating refractory IMDs,^44 45^ along with other B cell-targeted therapies such as rituximab, obinutuzumab and belimumab,^46 47^ may be further supported by the findings of the present study.

By comparing the immunophenotypes of our six IMDs with those of other IMDs, rather than healthy controls as in our previous work,^1^ we identified strong immunophenotypic characteristics that distinguished the unique attributes of these diseases. For instance, patients with SLE exhibited increased populations of Fr. III T, DN B and plasmablasts, while patients with SSc were characterised by an increased population of NC Mono. These findings resonate with prior comparative studies^48^,^51^ and highlight the utility of our approach in accentuating the immunophenotypic nuances among IMDs. Our analysis revealed that certain B-cell subsets, specifically USM B, displayed reduced populations in SLE compared with other IMDs, mirroring previous research.^49 52^ Furthermore, the inverse correlation between the USM B proportion and SLEDAI score,^52^ as well as the increased proportion of naïve B and decreased proportion of SM B in active SLE cases,^49^ align with our cluster 2. This reinforces the significant clinical relevance of these immunophenotypic alterations.

Our immunophenotyping-based classification aligns with findings by Barturen *et al,* who used integrative transcriptomic and methylome analyses to identify overarching ‘inflammatory’, ‘lymphoid’ and ‘interferon’ clusters that go beyond traditional diagnoses in systemic autoimmune diseases.^10^ Notably, Barturen *et al* showed that the ‘interferon’ cluster was associated with higher SLEDAI scores in patients with SLE, mirroring our observation that cluster 2—characterised by elevated type I IFN pathway interactions—likewise exhibited higher SLEDAI scores. Furthermore, our data suggest that shifts in B-cell subsets may underlie these transcriptional differences. Taken together, these findings underscore how multiparameter immune profiling can reveal convergent pathobiological mechanisms across otherwise distinct immune-mediated disorders, suggesting a potential framework for more tailored therapeutic strategies.

In cluster 1, a decreased %VC and increased prevalence of specific autoantibodies, such as anti-Scl-70 and anti-RNA polymerase III antibodies, was observed in patients with SSc. A previous study elucidated that patients with severe SSc-associated ILD had higher proportions of DN B cells, as well as CD21^lo/neg^ t-bet^+^ B cells.^53^ Intriguingly, the infiltration of CD21^lo/neg^ B cells into the lung parenchyma of these patients presents a novel aspect of disease pathology. Additionally, patients with RA who had higher disease activity were also included in cluster 1, which was associated with a higher proportion of DN B and plasmablasts. Deconvolution analysis of the RA synovium revealed that subgroups with more ABCs and plasmablasts exhibited higher disease activity.^54^ The increased proportion of differentiated B-cell subsets in cluster 1 may indicate that these subsets infiltrate damaged organs, such as the lung and synovium, in SSc and RA.

In contrast, cluster 2 was linked to higher disease activity in SLE. In the current study, transcriptomic analysis revealed elevated expression of *CXCR3*, *TBX21* and *ITGAX*, all of which are associated with ABC,^36^ in cluster 2. These findings suggest that bulk B-cell populations, particularly SM B, DN B and plasmablasts, in SLE contain cell subsets with ABC signatures, consistent with previous studies of SLE. Jenks *et al* demonstrated that ABCs are significantly expanded in patients with SLE and are associated with increased disease activity and autoantibody production.^48^ Additionally, patients with SLE with expanded ABC cell subsets exhibited nephrotic kidneys,^55^ suggesting severe organ damage in SLE.

Simultaneously, B-cell activation markers such as CD69 and CD86 were also enriched in B-cell subsets in cluster 2. This cluster included patients with SLE with higher disease activity and higher titre of anti-dsDNA antibody and anti-cardiolipin antibody. Previously, double-negative 2 B cells, which share ABC features and activated naïve B cells (CD69^+^CD86^+^) were reported to be elevated in SLE.^56^ Additionally, the proportion of activated naïve B cells was correlated with SLEDAI-2K and linked to anti-dsDNA antibody production in that study. This aligns with our current findings, where patients with SLE in cluster 2 exhibited a higher ABC and activated B-cell signature. This may also explain the higher IgG titres observed in six IMD patients in cluster 2. Future single-cell RNA sequencing studies may help clarify the trajectory of this potential ABC population.

Elevated oxidative phosphorylation scores were observed in USM B and DN B in cluster 1. Previously, we demonstrated that an oxidative phosphorylation signature in SLE was associated with a higher SDI score,^12^ highlighting mitochondrial dysfunction as a key immunological pathway influencing the long-term outcomes of SLE. Interestingly, despite no statistical significance (p=0.11), patients with SLE in cluster 1 exhibited a higher SDI in this study. In contrast, patients with SLE with a higher SLEDAI score were included in cluster 2. Cluster 2 demonstrated significantly higher mTORC1 signalling in DN B. Furthermore, DN B also showed a higher ABC signature in patients with SLE within cluster 2. mTORC1 modulates mitochondrial activity and promotes translation of nucleus-encoded mitochondria-related transcripts.^57^ In particular, CD11c^+^T-bet^+^CD21^−^ memory B cells, which are correlated with the SLEDAI, showed enrichment of the mTORC1 pathway.^58^ Although this study did not examine CD11c^+^T-bet^+^CD21^−^ memory B cells, these subsets might be included in DN B.^48^ Collectively, differences in mitochondrial function in the B-cell subsets of SLE may play an important role in high disease activity and long-term organ damage in distinct ways.

The upregulation of glycosylation-related genes observed in USM B within cluster 1 merits attention, given the emerging role of glycosylation in modulating the immune response in autoimmune diseases. Altered glycosylation patterns, such as changes in galactosylation and sialylation of antibodies, impact their effector functions and contribute to disease pathology.^59^ This is supported by the observed correlation between altered IgG glycosylation and disease activity in RA.^60^ In the current study, CDAI and SDAI scores were significantly higher in cluster 1, which was enriched in glycosylation pathways in USM B. Although the relationship between antibody or cell surface glycosylation and disease activity within SLE is still under debate, a study indicated a potential link between the structure of IgG N-glycans and inflammatory processes in patients with lupus nephritis.^61^ That study also suggested that variations in IgG sialylation, galactosylation, core fucosylation and bisecting N-acetylglucosamine could influence antibody function and may be involved in the development of lupus nephritis.^61^ This difference may contribute to the pathogenesis and progression of the disease.

Analysis of cell–cell communication revealed that DN B cells and plasmablasts, along with other B-cell subsets in cluster 1 may receive pronounced BAFF signalling from monocytes and DCs according to the ligand-receptor expression. Given the increased presence of mature B cells in cluster 1 and the enrichment of BAFF interactions, belimumab, a human monoclonal antibody that specifically binds to and inhibits BAFF, may represent a feasible therapeutic option.^62 63^ Indeed, alterations in the transcriptome of USM B indicate a good response to belimumab in patients with SLE.^33^ In contrast, we found that monocyte subsets, particularly Int Mono and NC Mono in cluster 2, exhibited higher IFN-α and IFN-γ responses, respectively. A similar trend of elevated type I IFN production by myeloid cells was observed in the cell–cell interaction analysis. This is concordant with a previous single-cell RNA sequencing analysis of paediatric lupus, revealing enriched IFN-stimulated genes, especially in both CD16^+^ and CD14^+^ monocytes, in patients with high disease activity,^64^ suggesting a pivotal role for monocytes as major IFN producers in active disease states. For patients classified in cluster 2, JAK inhibitors and anifrolumab, the latter of which targets type I IFN receptor subunit 1, may represent optimal treatment strategies. While differences in autoantibody profiles were evident only in SLE and SSc, variations in BAFF and type I IFN signalling pathways suggest alternative mechanisms influencing B-cell activation and disease progression.

Although MCTD was associated with a decreased proportion of USM B cells, similar to what is observed in SLE, we recently identified that MCTD can be further categorised into SLE-like, IIM-like and SSc-like immunophenotypes.^9^ Among these, the SLE-like immunophenotype was characterised by more SLE-related symptoms and elevated IFN signatures. These findings highlight the heterogeneity within MCTD and suggest that immunophenotyping can potentially be used to stratify patients and guide treatment approaches. In addition, we previously reported that increased muscle-infiltrating monocytes were associated with muscle damage in patients with IIM.^65^ In the present study using PBMCs, IIM did not demonstrate monocyte variation nor skewed cluster distribution. Therefore, immunophenotyping of affected organs might be useful to understand disease pathogenesis.

Striking disparities emerged in our previous analyses, revealing distinct immune alterations between each disease and healthy controls.^1^ In SLE, the number of DEGs between inactive SLE and healthy controls was particularly prominent in B-cell subsets,^33^ including naïve B, USM B, SM B and DN B, suggesting B-cell dysregulation even during the inactive disease phase. This finding further supports the crucial role of B-cell alterations in IMDs. Among patients with IIM, those with active IIM showed significantly decreased proportions of USM B and SM B compared with healthy controls.^5^ Notably, SM B cells were only decreased in IIM compared with other IMDs in this study. In addition, type I IFN-stimulated genes were enriched across all immune cell subsets. In SSc, an inflammatory gene module in NC Mono was identified,^13^ which showed an increased cell proportion compared with other IMDs in the present study. This module, which includes genes such as *CXCL8, FOS, JUN* and *NFKBIA*, was significantly upregulated in patients with SSc compared with healthy controls. Additionally, both type I and type II IFN signatures were elevated across all immune cell subsets. Likewise, among RA, type I and type II IFN response genes, along with IL-6/JAK/STAT3 signalling, were enriched in various immune cell subsets, including B cells and myeloid cells, compared with healthy controls.^66^ Overall, pronounced IFN signatures were observed relative to healthy controls in our previous studies. Although these observations are crucial for understanding disease pathogenesis, the current study further identified nuanced and deeper differences among IMDs. Notably, patients in cluster 2 demonstrated heightened IFN signatures, indicating a stronger association compared with cluster 1, along with an increased naïve B-cell proportion.

In a European cohort of patients with SLE, whole-blood transcriptomic analysis identified clusters such as ‘interferon/plasma cells’, ‘inflammation’ and ‘lymphocyte signaling’.^67^ Patients in the ‘interferon/plasma cells’ cluster had higher SLEDAI scores, which partially aligned with our findings in cluster 2, as the proportion of plasmablasts in cluster 2 was decreased in our study. Additionally, a large European cohort of patients with SSc revealed deregulated pathways, including type I IFN signalling and toll-like receptor cascades.^68^ While our transcriptomic analysis of SSc also identified dysregulated IFN pathways in cluster 2, where most patients with SSc were included, the immunophenotypic findings were not identical to ours. One possible explanation for these differences could be ethnic variations in immune response.

Interestingly, our current study demonstrated that patients in cluster 2, characterised by increased naïve B with an ABC signature, exhibited a higher flare rate. Recent evidence supports that expansions of naïve B and ABCs may act as adverse prognostic indicators across various IMDs. In RA, recent single-cell and bulk RNA sequencing analysis have shown that increases in naïve B, IgD^+^CD24^+^ B cell (naïve B-like cell) and CXCR3^+^ B cell (ABC-like cell) can precede flares and disease onset, reinforcing a mechanistic connection between these B subsets and disease exacerbation.^69^,^71^ Notably, synovial tissue enriched with ABCs and other immune cells correlates with heightened disease activity, suggesting that ABCs serve as central mediators driving synovial inflammation.^54^ Similar patterns have emerged in other autoimmune conditions. In SLE, higher proportions of naïve B cells have been associated with an increased risk of disease flare, whereas predominance of memory B cells correlates with reduced flare rates.^72^ Likewise, in IIM, clusters rich in naïve B frequently show elevated muscle damage markers, suggesting these cells may exacerbate tissue injury via enhanced antigen presentation and proinflammatory cytokine production.^73^ In SSc often exhibit expanded naive B cells with a concomitant reduction in memory B cells and plasmablasts, potentially amplifying autoantibody production and tissue pathology.^74^ Collectively, these findings reinforce that elevated levels of naïve B and ABCs not only signify ongoing immune dysregulation but also may serve as biomarkers of poor prognosis across a wide spectrum of autoimmune disorders.

We acknowledge several limitations in our study. However, the observation that B-cell proportions may predict transcriptomic changes suggests a potentially feasible approach for assessing disease conditions in patients with IMDs. A mechanistic study investigating the biological significance of glycosylation pathways and B-cell receptor signalling is warranted. Additionally, future research should incorporate single-cell RNA sequencing, which provides a more nuanced view of immune cell variations by integrating immunophenotyping with transcriptomic data. Furthermore, validation studies and longitudinal analyses will be essential to elucidate the clinical utility of immunophenotyping.

Our study revealed significant associations between immunophenotypic profiles and transcriptomic landscapes across IMDs. By analysing a large patient cohort, we found that B-cell subset heterogeneity offers insights into disease pathogenesis, potential treatment strategies and the prediction of disease flares, underscoring the value of integrating transcriptomics with immunophenotyping to understand immune status in IMDs.

## Supplementary material

10.1136/rmdopen-2024-005310 Supplementary Figure 1

10.1136/rmdopen-2024-005310 Supplementary Figure 2

10.1136/rmdopen-2024-005310 Supplementary Figure 3

10.1136/rmdopen-2024-005310 Supplementary Figure 4

10.1136/rmdopen-2024-005310 Supplementary Figure 5

10.1136/rmdopen-2024-005310 Supplementary Figure 6

10.1136/rmdopen-2024-005310 Supplementary Figure 7

10.1136/rmdopen-2024-005310 Supplementary Figure 8

10.1136/rmdopen-2024-005310 Supplementary Table 1

10.1136/rmdopen-2024-005310 Supplementary file 1

## Data Availability

Data are available in a public, open access repository. Data are available upon reasonable request.

## References

[1] Ota M, Nagafuchi Y, Hatano H (2021). Dynamic landscape of immune cell-specific gene regulation in immune-mediated diseases. Cell.

[2] Vital EM, Dass S, Buch MH (2011). B cell biomarkers of rituximab responses in systemic lupus erythematosus. Arthritis Rheum.

[3] Kubo S, Nakayamada S, Yoshikawa M (2017). Peripheral Immunophenotyping Identifies Three Subgroups Based on T Cell Heterogeneity in Lupus Patients. *Arthritis Rheumatol*.

[4] Wilkinson MGL, Radziszewska A, Wincup C (2020). Using peripheral blood immune signatures to stratify patients with adult and juvenile inflammatory myopathies. Rheumatology (Oxford).

[5] Sugimori Y, Iwasaki Y, Takeshima Y (2023). Transcriptome Profiling of Immune Cell Types in Peripheral Blood Reveals Common and Specific Pathways Involved in the Pathogenesis of Myositis-Specific Antibody-Positive Inflammatory Myopathies. *ACR Open Rheumatol*.

[6] Nagafuchi Y, Shoda H, Sumitomo S (2016). Immunophenotyping of rheumatoid arthritis reveals a linkage between HLA-DRB1 genotype, CXCR4 expression on memory CD4(+) T cells, and disease activity. Sci Rep.

[7] Kubo S, Miyazaki Y, Nishino T (2025). Peripheral blood immunophenotypic diversity in patients with rheumatoid arthritis and its impact on therapeutic responsiveness. Ann Rheum Dis.

[8] van der Kroef M, van den Hoogen LL, Mertens JS (2020). Cytometry by time of flight identifies distinct signatures in patients with systemic sclerosis, systemic lupus erythematosus and Sjögrens syndrome. Eur J Immunol.

[9] Izuka S, Komai T, Itamiya T (2025). Machine learning–driven immunophenotypic stratification of mixed connective tissue disease, corroborating the clinical heterogeneity. Rheumatology (Sunnyvale).

[10] Barturen G, Babaei S, Català-Moll F (2021). Integrative Analysis Reveals a Molecular Stratification of Systemic Autoimmune Diseases. *Arthritis Rheumatol*.

[11] Toro-Domínguez D, Martorell-Marugán J, Goldman D (2018). Stratification of Systemic Lupus Erythematosus Patients Into Three Groups of Disease Activity Progression According to Longitudinal Gene Expression. *Arthritis Rheumatol*.

[12] Takeshima Y, Iwasaki Y, Nakano M (2022). Immune cell multiomics analysis reveals contribution of oxidative phosphorylation to B-cell functions and organ damage of lupus. Ann Rheum Dis.

[13] Kobayashi S, Nagafuchi Y, Okubo M (2021). Integrated bulk and single-cell RNA-sequencing identified disease-relevant monocytes and a gene network module underlying systemic sclerosis. J Autoimmun.

[14] Tanaka H, Okada Y, Nakayamada S (2023). OP0001 DECONVOLUTING IMMUNOLOGICAL AND CLINICAL HETEROGENEITY ACROSS AUTOIMMUNE RHEUMATIC DISEASES BY COHORT-WIDE IMMUNO-PHENOTYPING. Ann Rheum Dis.

[15] Martin-Gutierrez L, Peng J, Thompson NL (2021). Stratification of Patients With Sjögren’s Syndrome and Patients With Systemic Lupus Erythematosus According to Two Shared Immune Cell Signatures, With Potential Therapeutic Implications. *Arthritis Rheumatol*.

[16] Robinson GA, Peng J, Dönnes P (2020). Disease-associated and patient-specific immune cell signatures in juvenile-onset systemic lupus erythematosus: patient stratification using a machine-learning approach. *Lancet Rheumatol*.

[17] Ye Y, Zhang X, Li T (2022). Two Distinct Immune Cell Signatures Predict the Clinical Outcomes in Patients With Amyopathic Dermatomyositis With Interstitial Lung Disease. *Arthritis Rheumatol*.

[18] Hochberg MC (1997). Updating the American College of Rheumatology revised criteria for the classification of systemic lupus erythematosus. Arthritis Rheum.

[19] Kasukawa R (1999). Mixed connective tissue disease. Intern Med.

[20] Bohan A, Peter JB (1975). Polymyositis and dermatomyositis (first of two parts). N Engl J Med.

[21] Bohan A, Peter JB (1975). Polymyositis and dermatomyositis (second of two parts). N Engl J Med.

[22] Hoogendijk JE, Amato AA, Lecky BR (2004). 119th ENMC international workshop: Trial design in adult idiopathic inflammatory myopathies, with the exception of inclusion body myositis, 10–12 October 2003, Naarden, The Netherlands. Neuromuscul Disord.

[23] Aletaha D, Neogi T, Silman AJ (2010). 2010 Rheumatoid arthritis classification criteria: an American College of Rheumatology/European League Against Rheumatism collaborative initiative. Arthritis Rheum.

[24] van den Hoogen F, Khanna D, Fransen J (2013). 2013 classification criteria for systemic sclerosis: an American College of Rheumatology/European League against Rheumatism collaborative initiative. Arthritis Rheum.

[25] Arend WP, Michel BA, Bloch DA (1990). The American College of Rheumatology 1990 criteria for the classification of Takayasu arteritis. Arthritis Rheum.

[26] Hunder GG, Bloch DA, Michel BA (1990). The American College of Rheumatology 1990 criteria for the classification of giant cell arteritis. Arthritis Rheum.

[27] Buyon JP, Petri MA, Kim MY (2005). The effect of combined estrogen and progesterone hormone replacement therapy on disease activity in systemic lupus erythematosus: a randomized trial. Ann Intern Med.

[28] Saygin D, Oddis CV, Marder G (2020). Follow-up results of myositis patients treated with H. P. Acthar gel. *Rheumatology (Oxford)*.

[29] van Leeuwen NM, Liem SIE, Maurits MP (2021). Disease progression in systemic sclerosis. Rheumatology (Oxford).

[30] Yazici Y, Erkan D, Kulman I (2002). Decreased flares of rheumatoid arthritis during the first year of etanercept treatment: further evidence of clinical effectiveness in the “real world”. Ann Rheum Dis.

[31] Hellmich B, Agueda A, Monti S (2020). 2018 Update of the EULAR recommendations for the management of large vessel vasculitis. Ann Rheum Dis.

[32] Hennig C (2007). Cluster-wise assessment of cluster stability. Computational Statistics & Data Analysis.

[33] Nakano M, Ota M, Takeshima Y (2022). Distinct transcriptome architectures underlying lupus establishment and exacerbation. Cell.

[34] Korthauer K, Kimes PK, Duvallet C (2019). A practical guide to methods controlling false discoveries in computational biology. Genome Biol.

[35] Liberzon A, Birger C, Thorvaldsdóttir H (2015). The Molecular Signatures Database (MSigDB) hallmark gene set collection. Cell Syst.

[36] Sutton HJ, Aye R, Idris AH (2021). Atypical B cells are part of an alternative lineage of B cells that participates in responses to vaccination and infection in humans. Cell Rep.

[37] Espinoza DA, Le Coz C, Cruz Cabrera E (2023). Distinct stage-specific transcriptional states of B cells derived from human tonsillar tissue. JCI Insight.

[38] Reyes RA, Batugedara G, Dutta P (2023). Atypical B cells consist of subsets with distinct functional profiles. iScience.

[39] Hao Y, Stuart T, Kowalski MH (2024). Dictionary learning for integrative, multimodal and scalable single-cell analysis. Nat Biotechnol.

[40] Piga M, Parodis I, Touma Z (2025). Framework for implementing treat-to-target in systemic lupus erythematosus routine clinical care: consensus statements from an international task force. Autoimmun Rev.

[41] Song W, Antao OQ, Condiff E (2022). Development of Tbet- and CD11c-expressing B cells in a viral infection requires T follicular helper cells outside of germinal centers. Immunity.

[42] Brown GJ, Cañete PF, Wang H (2022). TLR7 gain-of-function genetic variation causes human lupus. Nature New Biol.

[43] Dai D, Gu S, Han X (2024). The transcription factor ZEB2 drives the formation of age-associated B cells. Science.

[44] Schett G, Müller F, Taubmann J (2024). Advancements and challenges in CAR T cell therapy in autoimmune diseases. Nat Rev Rheumatol.

[45] Schett G, Nagy G, Krönke G (2024). B-cell depletion in autoimmune diseases. Ann Rheum Dis.

[46] Fanouriakis A, Kostopoulou M, Andersen J (2024). EULAR recommendations for the management of systemic lupus erythematosus: 2023 update. Ann Rheum Dis.

[47] Furie RA, Aroca G, Cascino MD (2022). B-cell depletion with obinutuzumab for the treatment of proliferative lupus nephritis: a randomised, double-blind, placebo-controlled trial. Ann Rheum Dis.

[48] Jenks SA, Cashman KS, Zumaquero E (2018). Distinct Effector B Cells Induced by Unregulated Toll-like Receptor 7 Contribute to Pathogenic Responses in Systemic Lupus Erythematosus. Immunity.

[49] Rodríguez-Bayona B, Ramos-Amaya A, Pérez-Venegas JJ (2010). Decreased frequency and activated phenotype of blood CD27 IgD IgM B lymphocytes is a permanent abnormality in systemic lupus erythematosus patients. Arthritis Res Ther.

[50] Schneider L, Marcondes NA, Hax V (2021). Flow cytometry evaluation of CD14/CD16 monocyte subpopulations in systemic sclerosis patients: a cross sectional controlled study. *Adv Rheumatol*.

[51] Matei A-E, Kubánková M, Xu L (2023). Identification of a Distinct Monocyte-Driven Signature in Systemic Sclerosis Using Biophysical Phenotyping of Circulating Immune Cells. *Arthritis Rheumatol*.

[52] Zhang W, Wang Y-F, Hu F-L (2022). Dysfunction of CD27+IgD+ B cells correlates with aggravated systemic lupus erythematosus. Clin Rheumatol.

[53] Wilfong EM, Vowell KN, Bunn KE (2022). CD19 + CD21^lo/neg^ cells are increased in systemic sclerosis-associated interstitial lung disease. Clin Exp Med.

[54] Nakajima S, Tsuchiya H, Ota M (2023). Synovial Tissue Heterogeneity in Japanese Patients With Rheumatoid Arthritis Elucidated Using a Cell-Type Deconvolution Approach. *Arthritis Rheumatol*.

[55] Wang S, Wang J, Kumar V (2018). IL-21 drives expansion and plasma cell differentiation of autoreactive CD11chiT-bet+ B cells in SLE. Nat Commun.

[56] Wangriatisak K, Thanadetsuntorn C, Krittayapoositpot T (2021). The expansion of activated naive DNA autoreactive B cells and its association with disease activity in systemic lupus erythematosus patients. Arthritis Res Ther.

[57] Morita M, Gravel S-P, Chénard V (2013). mTORC1 controls mitochondrial activity and biogenesis through 4E-BP-dependent translational regulation. Cell Metab.

[58] Wu C, Fu Q, Guo Q (2019). Lupus-associated atypical memory B cells are mTORC1-hyperactivated and functionally dysregulated. Ann Rheum Dis.

[59] Kissel T, Toes REM, Huizinga TWJ (2023). Glycobiology of rheumatic diseases. Nat Rev Rheumatol.

[60] Ercan A, Cui J, Chatterton DEW (2010). Aberrant IgG galactosylation precedes disease onset, correlates with disease activity, and is prevalent in autoantibodies in rheumatoid arthritis. Arthritis Rheum.

[61] Lu X, Wang L, Wang M (2023). Association between immunoglobulin G N-glycosylation and lupus nephritis in female patients with systemic lupus erythematosus: a case-control study. Front Immunol.

[62] Baker T, Sharifian H, Newcombe PJ (2024). Type I interferon blockade with anifrolumab in patients with systemic lupus erythematosus modulates key immunopathological pathways in a gene expression and proteomic analysis of two phase 3 trials. Ann Rheum Dis.

[63] Virtanen A, Spinelli FR, Telliez JB (2024). JAK inhibitor selectivity: new opportunities, better drugs?. *Nat Rev Rheumatol*.

[64] Nehar-Belaid D, Hong S, Marches R (2020). Mapping systemic lupus erythematosus heterogeneity at the single-cell level. Nat Immunol.

[65] Izuka S, Umezawa N, Komai T (2025). Muscle Tissue Transcriptome of Idiopathic Inflammatory Myopathy Reflects the Muscle Damage Process by Monocytes and Presence of Skin Lesions. *Arthritis Rheumatol*.

[66] Yamada S, Nagafuchi Y, Wang M (2023). Immunomics analysis of rheumatoid arthritis identified precursor dendritic cells as a key cell subset of treatment resistance. Ann Rheum Dis.

[67] Lindblom J, Toro-Domínguez D, Carnero-Montoro E (2023). Distinct gene dysregulation patterns herald precision medicine potentiality in systemic lupus erythematosus. J Autoimmun.

[68] Beretta L, Barturen G, Vigone B (2020). Genome-wide whole blood transcriptome profiling in a large European cohort of systemic sclerosis patients. Ann Rheum Dis.

[69] Orange DE, Yao V, Sawicka K (2020). RNA Identification of PRIME Cells Predicting Rheumatoid Arthritis Flares. N Engl J Med.

[70] Baker KF, McDonald D, Hulme G (2024). Single-cell insights into immune dysregulation in rheumatoid arthritis flare versus drug-free remission. Nat Commun.

[71] Takada H, Demoruelle MK, Deane KD (2024). Expansion of HLA-DR Positive Peripheral Helper T and Naive B Cells in Anticitrullinated Protein Antibody-Positive Individuals At Risk for Rheumatoid Arthritis. *Arthritis Rheumatol*.

[72] Zheng J, Zhu L, Ju B (2022). Peripheral immunophenotypes associated with the flare in the systemic lupus erythematosus patients with low disease activity state. Clin Immunol.

[73] Pan Z, Li M, Zhang P (2025). Peripheral Blood Lymphocyte Subsets and Heterogeneity of B Cell Subsets in Patients of Idiopathic Inflammatory Myositis with Different Myositis-specific Autoantibodies. Inflammation.

[74] Beesley CF, Goldman NR, Taher TE (2022). Dysregulated B cell function and disease pathogenesis in systemic sclerosis. Front Immunol.

