# Supplementary figures and images for: Integration of transcriptome and immunophenotyping data highlights differences in the pathogenetic kinetics of B cells across immune-mediated disease
Source: RMD Open. 2025 Apr 9;11(2):e005310. doi: 10.1136/rmdopen-2024-005310 (PMC11987131; doi:10.1136/rmdopen-2024-005310)

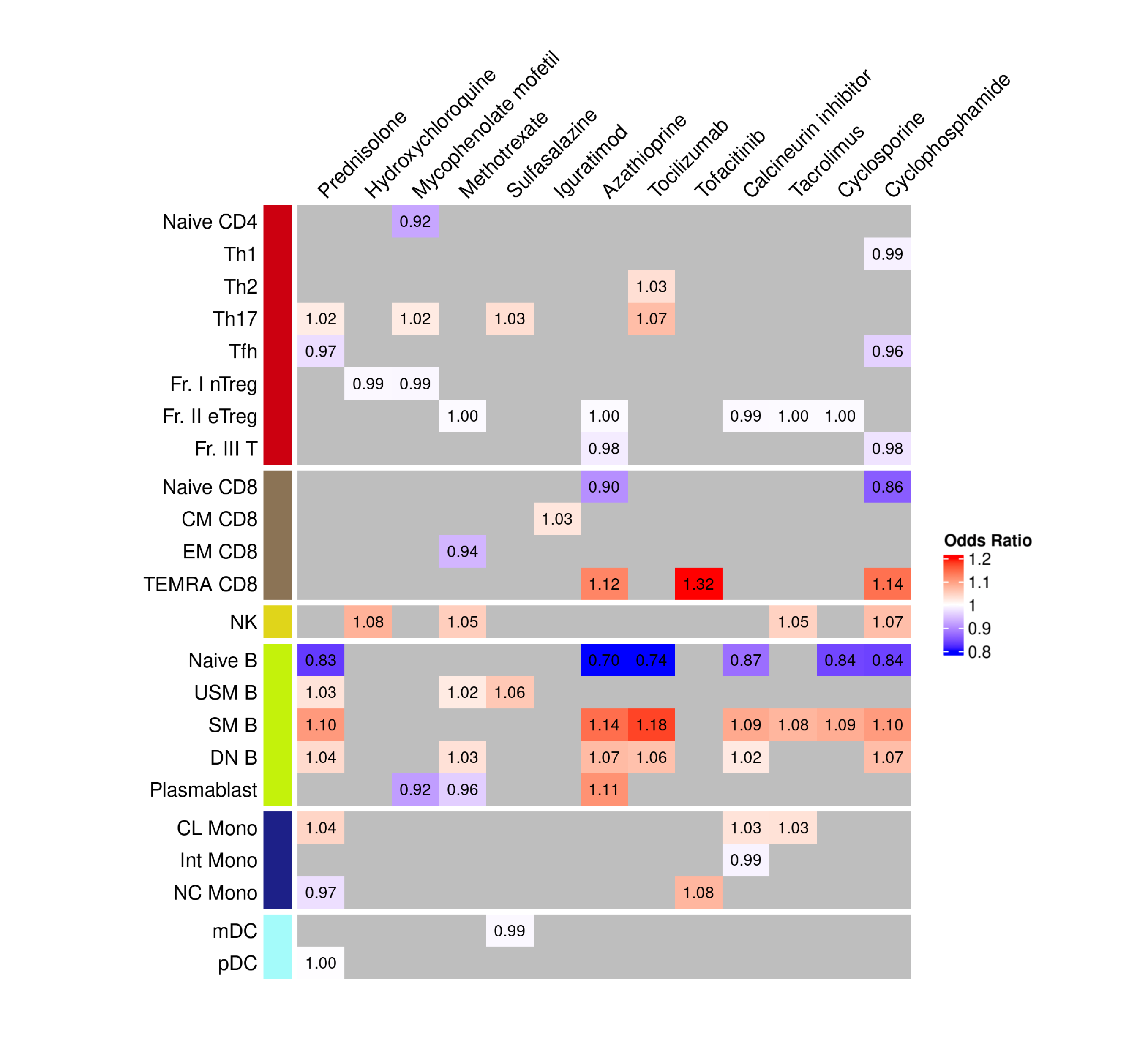

Supplement: Supplementary Figure 1 [file rmdopen-11-2-s001.tiff]

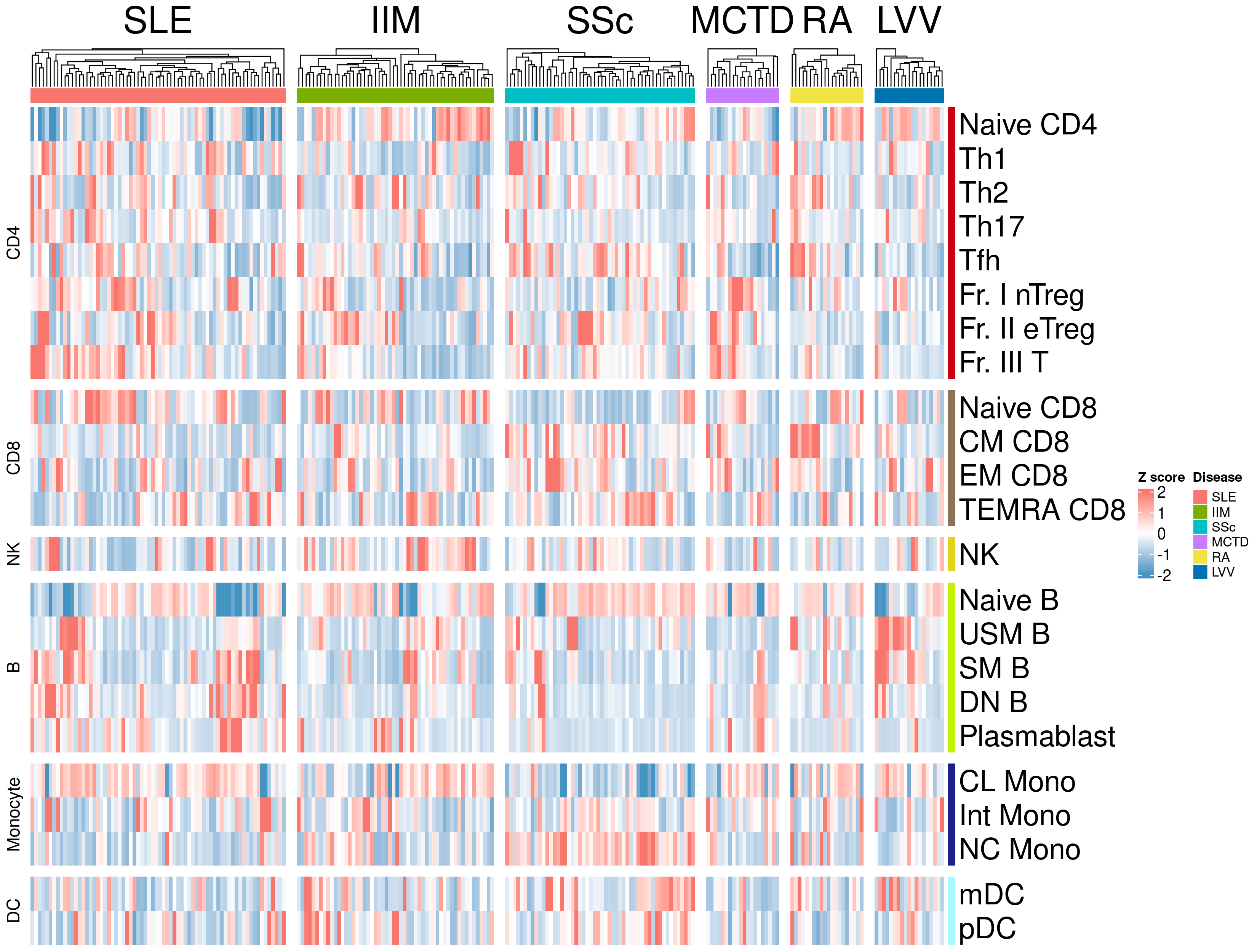

Supplement: Supplementary Figure 2 [file rmdopen-11-2-s002.tiff]

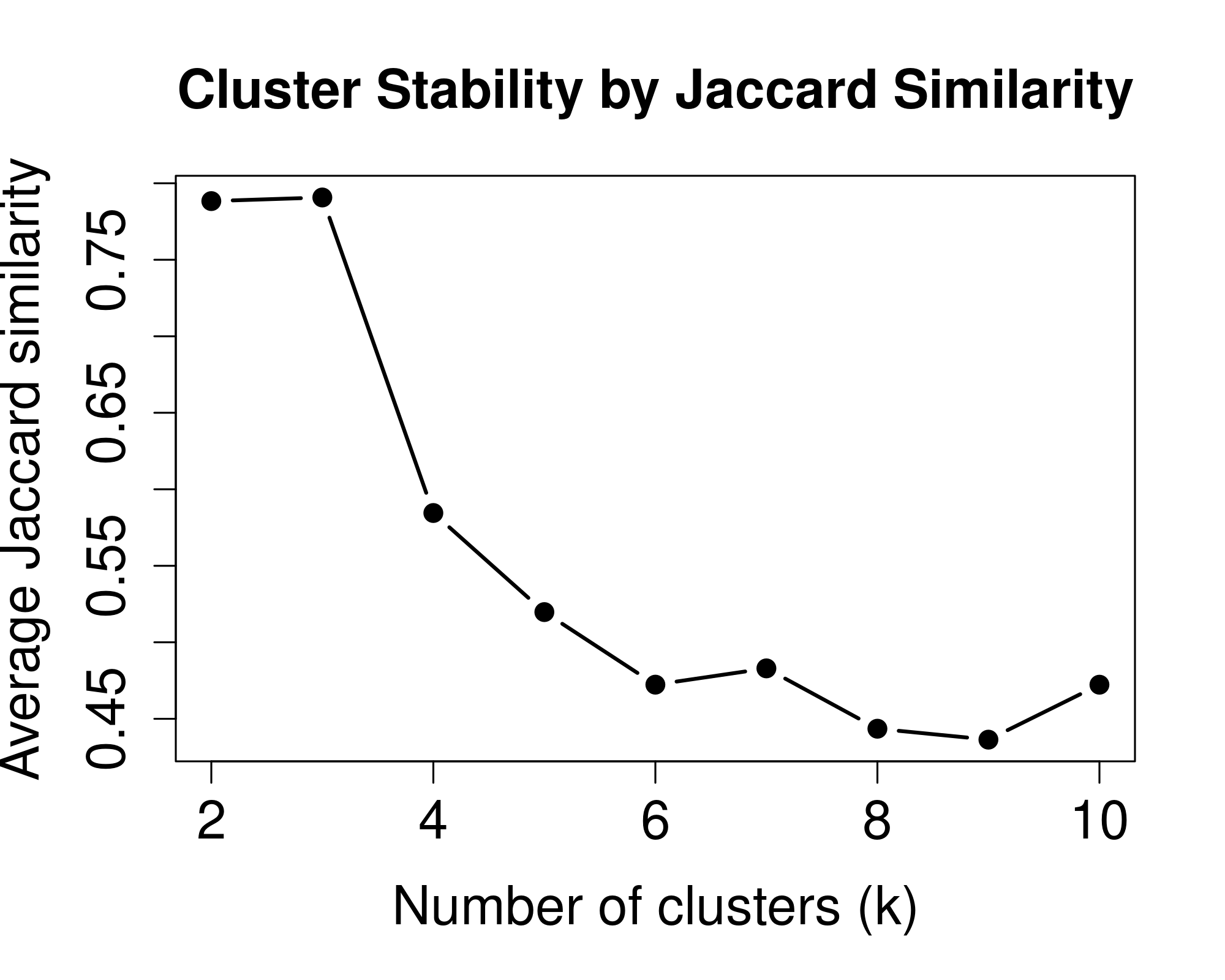

Supplement: Supplementary Figure 3 [file rmdopen-11-2-s003.tiff]

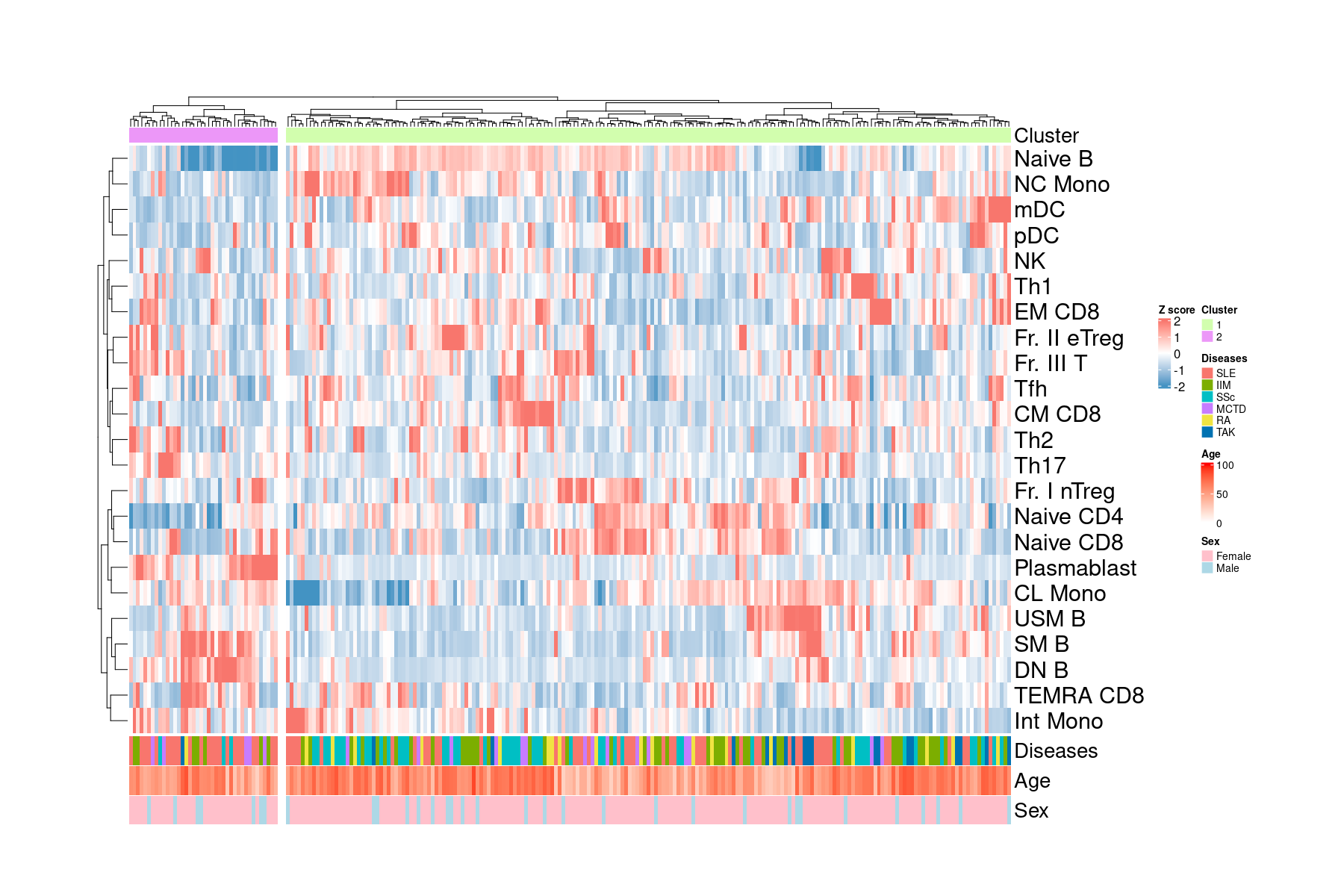

Supplement: Supplementary Figure 4 [file rmdopen-11-2-s004.tiff]

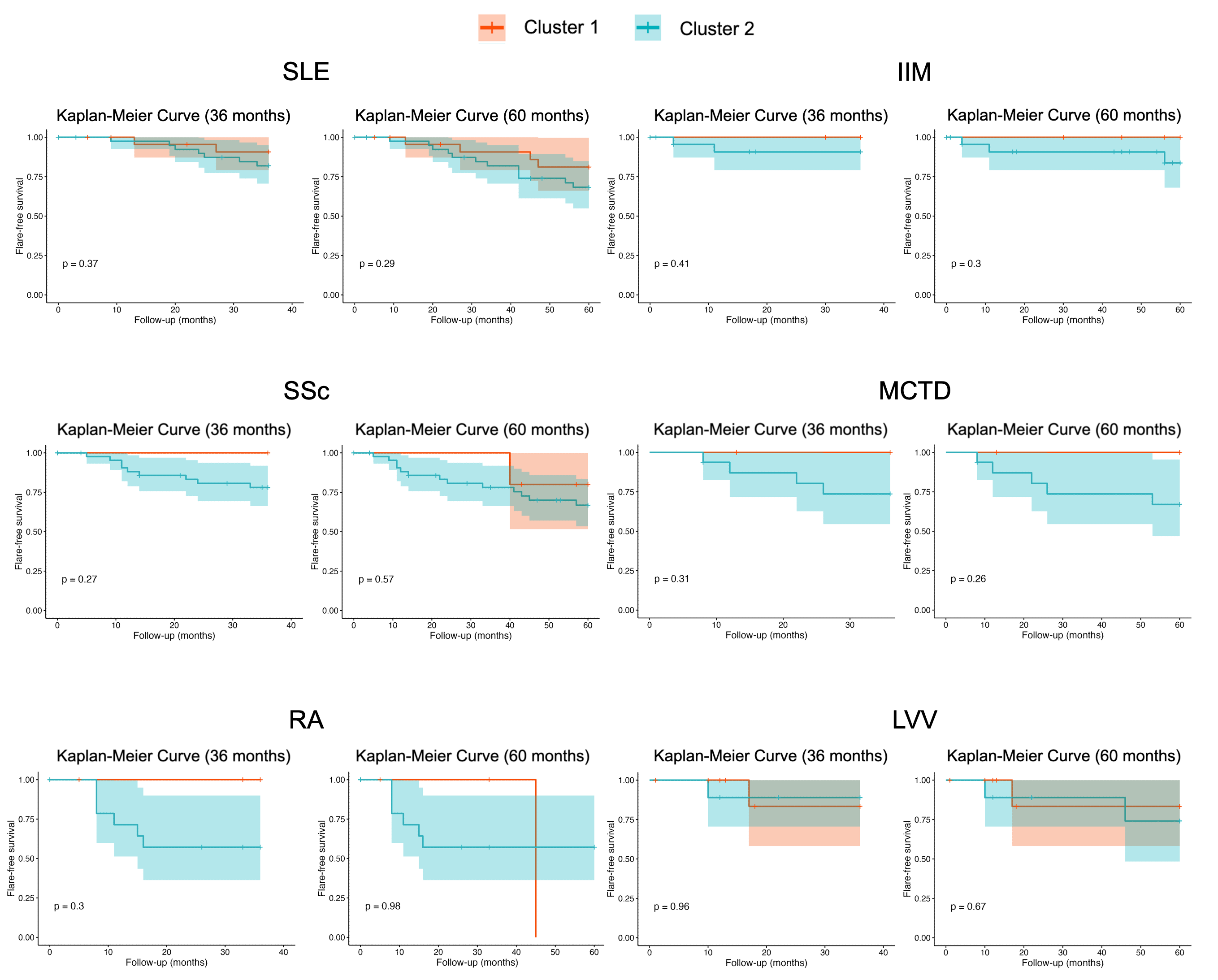

Supplement: Supplementary Figure 5 [file rmdopen-11-2-s005.tiff]

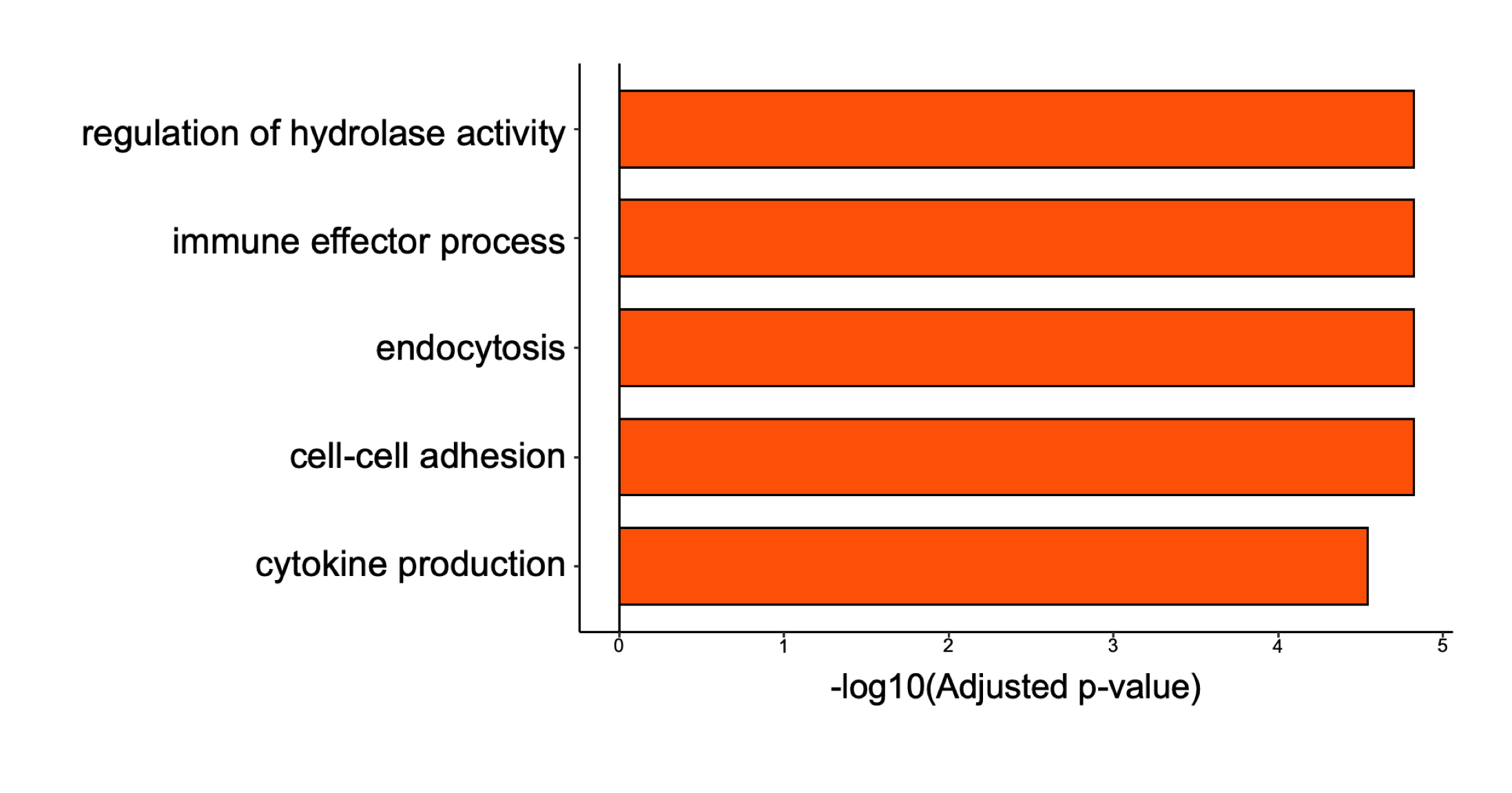

Supplement: Supplementary Figure 6 [file rmdopen-11-2-s006.tiff]

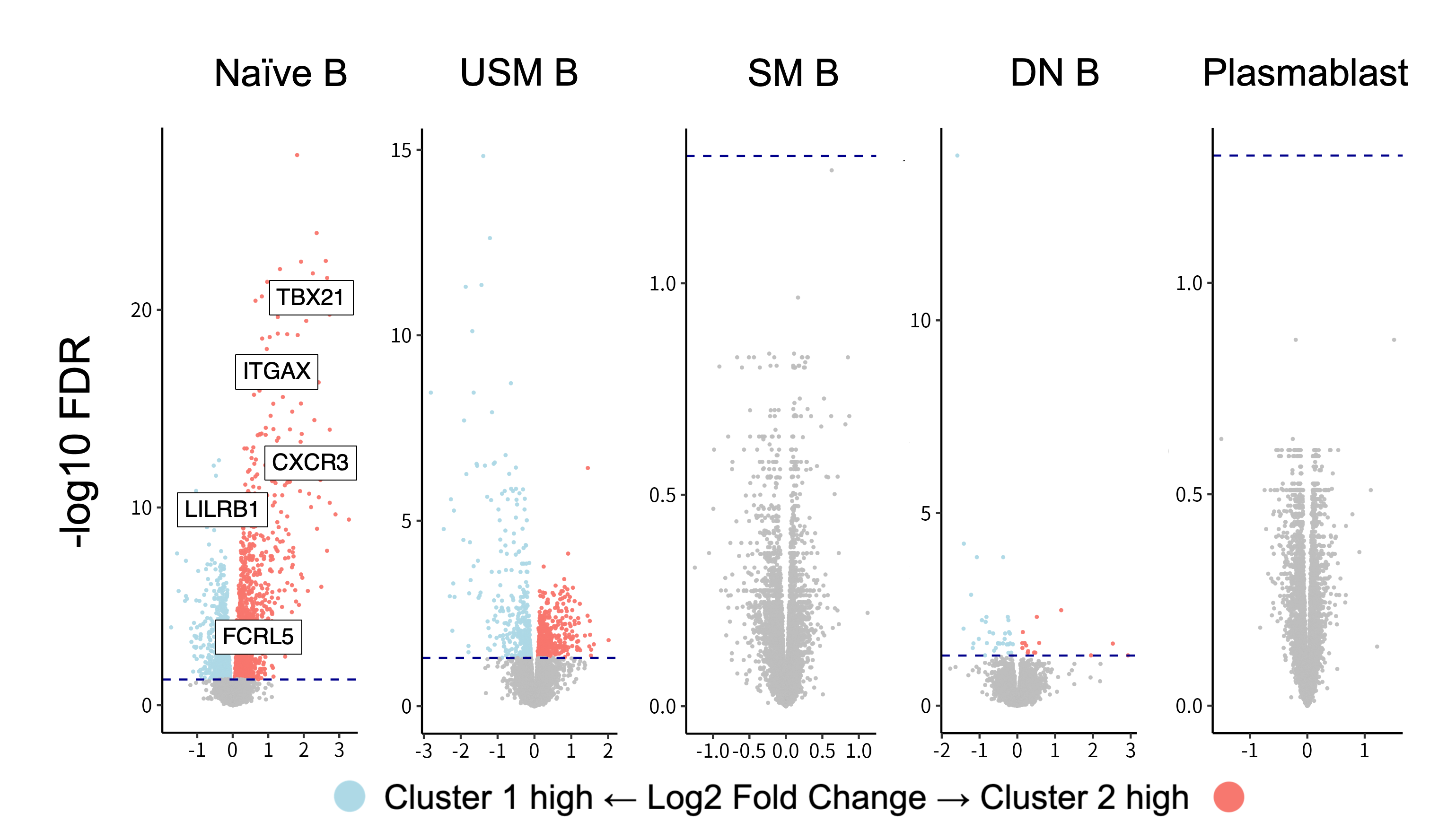

Supplement: Supplementary Figure 7 [file rmdopen-11-2-s007.tiff]

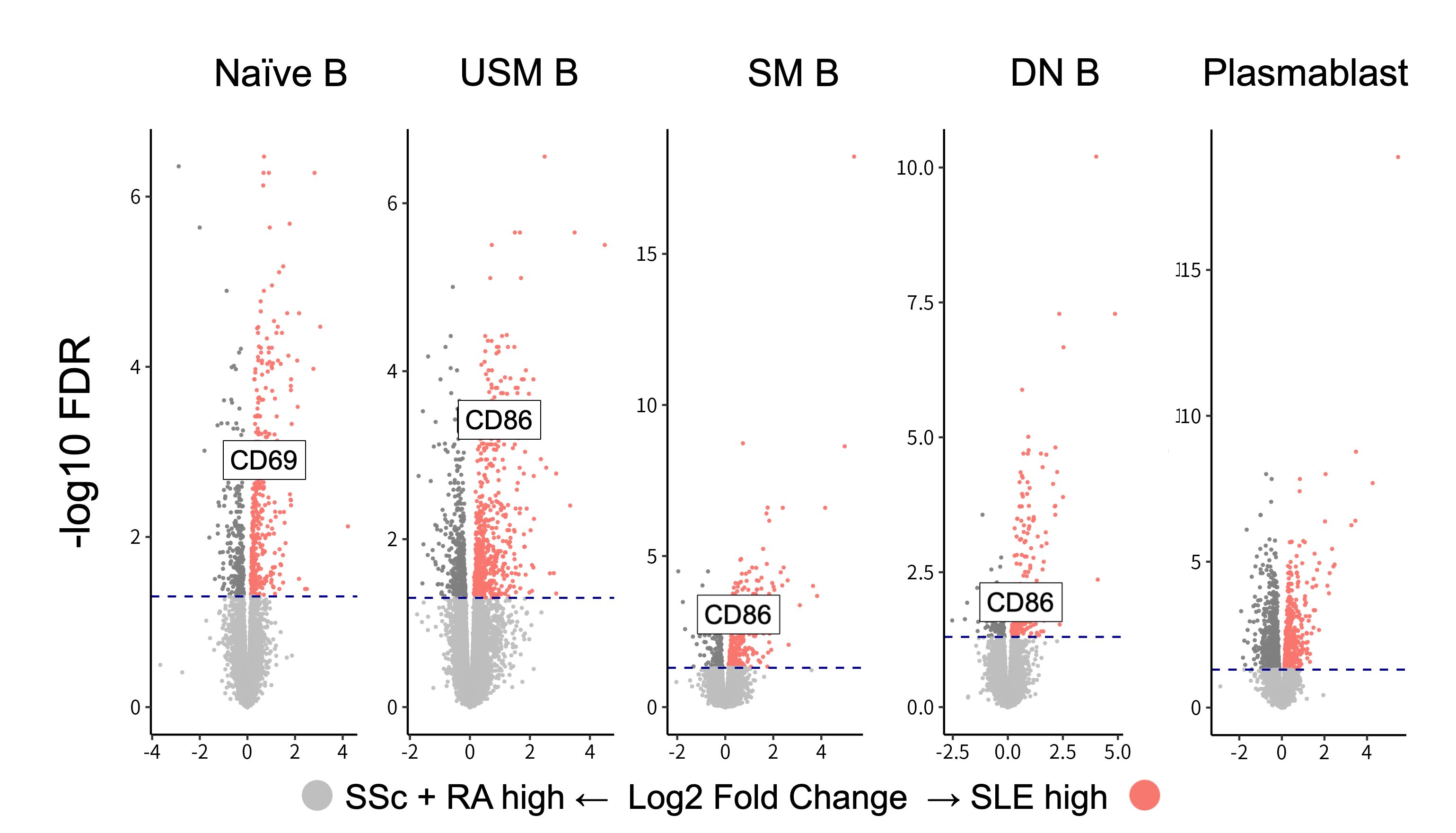

Supplement: Supplementary Figure 8 [file rmdopen-11-2-s008.tiff]
