# Supplementary material for: Integration of transcriptome and immunophenotyping data highlights differences in the pathogenetic kinetics of B cells across immune-mediated disease
Source: RMD Open. 2025 Apr 9;11(2):e005310. doi: 10.1136/rmdopen-2024-005310 (PMC11987131; doi:10.1136/rmdopen-2024-005310)
Supplement: Supplementary Table 1 [file rmdopen-11-2-s009.docx]

**Supplementary Table 1. Gating strategy**

| **Subset** | **Definition** |
| --- | --- |
| **Naive CD4** | CD3+/CD4+CD8-/CCR7+CD45RA+ |
| **Mem CD4** | CD3+/CD4+CD8-/non-naive CD4+/CD25- |
| **Th1** | CD3+/CD4+CD8-/non-naive CD4+/CD25-/CXCR5-CCR6-/CXCR3+CCR4- |
| **Th2** | CD3+/CD4+CD8-/non-naive CD4+/CD25-/CXCR5-CCR6-/CXCR3-CCR4+ |
| **Th17** | CD3+/CD4+CD8-/non-naive CD4+/CD25-/CXCR5-CCR6+/CXCR3- |
| **Tfh** | CD3+/CD4+CD8-/non-naive CD4+/CD25-/CXCR5+ |
| **Fr. I nTreg** | CD3+/CD4+CD8-/CD25+CD45RA+ |
| **Fr. II eTreg** | CD3+/CD4+CD8-/CD25++CD45RA- |
| **Fr. III T** | CD3+/CD4+CD8-/CD25+CD45RA- |
| **Naive CD8** | CD3+CD19-/CD4-CD8+/CD45RA+CCR7+ |
| **CM CD8** | CD3+CD19-/CD4-CD8+/CD45RA-CCR7+ |
| **EM CD8** | CD3+CD19-/CD4-CD8+/CD45RA-CCR7- |
| **TEMRA CD8** | CD3+CD19-/CD4-CD8+/CD45RA+CCR7- |
| **NK** | CD3-CD19-/CD14-/CD56+ |
| **Naive B** | CD3-CD19+/IgD+CD27- |
| **USM B** | CD3-CD19+/IgD+CD27+ |
| **SM B** | CD3-CD19+/IgD-CD27+/CD38- |
| **DN B** | CD3-CD19+/IgD-CD27- |
| **Plasmablast** | CD3-CD19+/IgD-CD27++/CD38+ |
| **CL Mono** | CD3-CD19-/HLADR+/CD56-/CD14+CD16- |
| **Int Mono** | CD3-CD19-/HLADR+/CD56-/CD14++CD16+ |
| **NC Mono** | CD3-CD19-/HLADR+/CD56-/CD14dimCD16+ |
| **mDC** | CD3-CD19-/HLADR+/CD56-/CD14-CD16-/CD11c+CD123- |
| **pDC** | CD3-CD19-/HLADR+/CD56-/CD14-CD16-/CD11c-CD123+ |

**Supplementary Table 2. Cell subset definitions**

| **Subset** | **Target** | **Parent** |
| --- | --- | --- |
| **Naive CD4** | CD4_Lymphocyte_CD3_CD4_NCD4 | CD4_Lymphocyte_CD3_CD4 |
| **Mem CD4** | CD4_Lymphocyte_CD3_CD4_MCD4 | CD4_Lymphocyte_CD3_CD4 |
| **Th1** | CD4_Lymphocyte_CD3_CD4_aTreg | CD4_Lymphocyte_CD3_CD4 |
| **Th2** | CD4_Lymphocyte_CD3_CD4_MCD4_Th1 | CD4_Lymphocyte_CD3_CD4 |
| **Th17** | CD4_Lymphocyte_CD3_CD4_MCD4_Th2 | CD4_Lymphocyte_CD3_CD4 |
| **Tfh** | CD4_Lymphocyte_CD3_CD4_MCD4_TH17 | CD4_Lymphocyte_CD3_CD4 |
| **Fr. I nTreg** | CD4_Lymphocyte_CD3_CD4_MCD4_Tfh | CD4_Lymphocyte_CD3_CD4 |
| **Fr. II eTreg** | CD4_Lymphocyte_CD3_CD4_Fra1 | CD4_Lymphocyte_CD3_CD4 |
| **Fr. III T** | CD4_Lymphocyte_CD3_CD4_Fra3 | CD4_Lymphocyte_CD3_CD4 |
| **Naive CD8** | BCD8_Lymphocyte_CD3_CD8_NCD8 | BCD8_Lymphocyte_CD3_CD8 |
| **CM CD8** | BCD8_Lymphocyte_CD3_CD8_CmCD8 | BCD8_Lymphocyte_CD3_CD8 |
| **EM CD8** | BCD8_Lymphocyte_CD3_CD8_EmCD8 | BCD8_Lymphocyte_CD3_CD8 |
| **TEMRA CD8** | BCD8_Lymphocyte_CD3_CD8_EffectorCD8 | BCD8_Lymphocyte_CD3_CD8 |
| **NK** | NK_Lymphocyte_NK | NK_Lymphocyte |
| **Naive B** | BCD8_Lymphocyte_CD19_NaiB | BCD8_Lymphocyte_CD19 |
| **USM B** | BCD8_Lymphocyte_CD19_UnswMB | BCD8_Lymphocyte_CD19 |
| **SM B** | BCD8_Lymphocyte_CD19_PBSwi_SwiMB | BCD8_Lymphocyte_CD19 |
| **DN B** | BCD8_Lymphocyte_CD19_DNB | BCD8_Lymphocyte_CD19 |
| **Plasmablast** | BCD8_Lymphocyte_CD19_PBSwi_PB | BCD8_Lymphocyte_CD19 |
| **CL Mono** | NK_LymphocyteMonocyte_HLADRposi_nonNK_CD16nMo | NK_LymphocyteMonocyte_HLADRposi_nonNK |
| **Int Mono** | NK_LymphocyteMonocyte_HLADRposi_nonNK_Intermediate | NK_LymphocyteMonocyte_HLADRposi_nonNK |
| **NC Mono** | NK_LymphocyteMonocyte_HLADRposi_nonNK_NonClassical | NK_LymphocyteMonocyte_HLADRposi_nonNK |
| **mDC** | NK_LymphocyteMonocyte_HLADRposi_nonNK_DCs_mDC | NK_LymphocyteMonocyte_HLADRposi_nonNK |
| **pDC** | NK_LymphocyteMonocyte_HLADRposi_nonNK_DCs_pDC | NK_LymphocyteMonocyte_HLADRposi_nonNK |

**Table S3. Clinical features of the patients with each immune-mediated disease**

|  | **SLE (n=70)** | **IIM (n=54)** | **SSc (n=52)** | **MCTD (n=20)** | **RA (n=20)** | **LVV (n=19)** |
| --- | --- | --- | --- | --- | --- | --- |
| **Male sex (%)** | 9 (12.9) | 13 (24.1) | 3 (5.8) | 4 (20.0) | 1 (5.0) | 3 (15.8) |
| **Age, median [IQR] years** | 47.00 [39.25, 56.75] | 58.00 [52.00, 69.00] | 67.50 [56.75, 73.25] | 51.50 [42.75, 67.00] | 63.00 [54.00, 71.50] | 62.00 [41.50, 73.50] |
| **Disease duration, median [IQR] years** | 12.00 [4.50, 20.00] | 3.00 [0.00, 13.50] | 15.50 [7.00, 26.50] | 17.50 [13.50, 22.25] | 12.00 [0.95, 23.10] | 22.00 [8.50, 31.00] |
| **C-reactive protein, median, mg/dL, median [IQR]** | 0.07 [0.03, 0.27] | 0.08 [0.04, 0.28] | 0.08 [0.03, 0.14] | 0.11 [0.05, 0.39] | 1.05 [0.54, 3.92] | 0.16 [0.06, 0.28] |
| **Erythrocyte sedimentation rate, mm/hr, median [IQR]** | 23.00 [12.00, 43.00] | 18.00 [7.75, 42.25] | 15.00 [11.00, 22.00] | 21.00 [13.00, 45.50] | 46.00 [21.25, 72.25] | 21.00 [7.00, 27.50] |
| **IgG, mg/dL, median, [IQR]** | 1461.00 [1127.00, 1872.00] | 1341.00 [1005.50, 1743.00] | 1452.00 [1118.50, 1680.00] | 1666.50 [1407.50, 2043.25] | 1386.00 [1091.00, 1557.75] | 933.00 [862.00, 1329.00] |
| **Treatment** |  |  |  |  |  |  |
| **Dose of prednisolone, mg, median, [IQR]** | 5.00 [2.50, 7.00] | 3.00 [0.00, 6.00] | 0.00 [0.00, 0.00] | 5.00 [4.00, 6.62] | 2.50 [0.00, 5.00] | 5.00 [3.00, 7.25] |
| **Hydroxychloroquine (%)** | 25 (35.7) | 0 (0.0) | 0 (0.0) | 1 (5.0) | 0 (0.0) | 0 (0.0) |
| **Tacrolimus (%)** | 9 (12.9) | 11 (17.5) | 4 (7.7) | 2 (10.0) | 1 (5.0) | 2 (10.5) |
| **Cyclosporine A (%)** | 7 (10.0) | 4 (7.4) | 1 (1.9) | 1 (5.0) | 0 (0.0) | 1 (5.3) |
| **Mycophenolate mofetil (%)** | 10 (14.3) | 0 (0.0) | 3 (5.8) | 0 (0.0) | 0 (0.0) | 0 (0.0) |
| **Azathioprine (%)** | 9 (12.9) | 1 (1.9) | 1 (1.9) | 3 (15.0) | 0 (0.0) | 3 (15.8) |
| **Methotrexate (%)** | 3 (4.3) | 9 (16.7) | 4 (7.7) | 2 (10.0) | 7 (35.0) | 4 (21.1) |

IQR = interquartile range

**Supplementary Table 4. Absolute values from principal component analysis of the two clusters**

| **Subset** | **PC1** | **PC2** | **PC3** | **PC4** | **PC5** |
| --- | --- | --- | --- | --- | --- |
| **Naive CD4** | **-0.17185267** | **0.459317744** | **-0.03801404** | **0.04259435** | **-0.02261745** |
| **Th1** | **0.05989828** | **-0.265203355** | **0.14183885** | **0.18693171** | **0.2122801** |
| **Th2** | **-0.04308582** | **-0.232273963** | **0.21892088** | **-0.14409645** | **-0.18259249** |
| **Th17** | **0.104125** | **-0.21765084** | **0.19395223** | **-0.01672583** | **-0.12434776** |
| **Tfh** | **-0.08281974** | **-0.335944216** | **0.19854565** | **0.13879906** | **-0.12746878** |
| **Fr. I nTreg** | **-0.03212968** | **0.177790314** | **0.16790163** | **-0.06515546** | **-0.38553707** |
| **Fr. II eTreg** | **-0.09785371** | **-0.187176461** | **0.12050755** | **-0.35209725** | **-0.0526488** |
| **Fr. III T** | **0.02708853** | **-0.238719041** | **0.28357174** | **-0.19057622** | **-0.33182109** |
| **Naive CD8** | **-0.05967125** | **0.400686764** | **0.22104556** | **-0.05754075** | **-0.2551494** |
| **CM CD8** | **-0.11979116** | **-0.193642728** | **0.21636572** | **0.21879687** | **-0.10219182** |
| **EM CD8** | **-0.08298172** | **-0.237093981** | **0.0379294** | **0.17066588** | **0.20025059** |
| **TEMRA CD8** | **0.14244598** | **-0.07986996** | **-0.34406772** | **-0.16069438** | **0.17758872** |
| **NK** | **0.07859167** | **-0.092857568** | **0.01849407** | **-0.02873891** | **0.28640262** |
| **Naive B** | **-0.46753545** | **0.027805556** | **0.12867131** | **-0.01490518** | **0.25073896** |
| **USM B** | **0.24676514** | **0.021718861** | **-0.08505047** | **0.39307141** | **-0.15145029** |
| **SM B** | **0.41113574** | **-0.035005012** | **-0.16644382** | **0.18153399** | **-0.21028404** |
| **DN B** | **0.36868883** | **-0.104515561** | **-0.18537077** | **-0.0617404** | **-0.07998755** |
| **Plasmablast** | **0.22884669** | **0.044722463** | **0.04457997** | **-0.34548386** | **-0.1422957** |
| **CL Mono** | **0.29418721** | **0.216121** | **0.40002774** | **0.04043823** | **0.26335934** |
| **Int Mono** | **-0.09755247** | **-0.099553213** | **-0.34123649** | **-0.33586223** | **-0.15600681** |
| **NC Mono** | **-0.31022206** | **-0.193016956** | **-0.35504377** | **0.04332072** | **-0.14593595** |
| **mDC** | **-0.09022103** | **-0.008668018** | **-0.06773245** | **0.44748249** | **-0.26467163** |
| **pDC** | **-0.21784947** | **0.047699969** | **-0.13984478** | **0.15501371** | **-0.23860588** |

**Supplementary Table 5. Immunophenotypes of the two clusters**

|  | **Cluster 1 (n=66)** | **Cluster 2 (n=169)** | **Adjusted p-value** |
| --- | --- | --- | --- |
| **CD4 T cells:** |  |  |  |
| **Naive CD4, median [IQR]** | 0.29 [0.20, 0.39] | 0.34 [0.27, 0.43] | 0.021 |
| **Th1, median [IQR]** | 0.02 [0.01, 0.03] | 0.02 [0.02, 0.03] | 0.99 |
| **Th2, median [IQR]** | 0.03 [0.02, 0.04] | 0.03 [0.02, 0.04] | 0.99 |
| **Th17, median [IQR]** | 0.04 [0.03, 0.05] | 0.03 [0.03, 0.04] | 0.069 |
| **Tfh, median [IQR]** | 0.09 [0.06, 0.11] | 0.10 [0.08, 0.13] | 0.27 |
| **Fr. I nTreg, median [IQR]** | 0.02 [0.02, 0.02] | 0.02 [0.01, 0.03] | 0.99 |
| **Fr. II eTreg, median [IQR]** | 0.01 [0.01, 0.01] | 0.01 [0.01, 0.01] | 0.99 |
| **Fr. III T, median [IQR]** | 0.04 [0.03, 0.06] | 0.05 [0.03, 0.06] | 0.99 |
| **CD8 T cells:** |  |  |  |
| **Naive CD8, median [IQR]** | 0.25 [0.12, 0.35] | 0.26 [0.16, 0.40] | 0.99 |
| **CM CD8, median [IQR]** | 0.03 [0.02, 0.04] | 0.04 [0.03, 0.05] | 0.024 |
| **EM CD8, median [IQR]** | 0.13 [0.10, 0.17] | 0.16 [0.11, 0.21] | 0.15 |
| **TEMRA CD8, median [IQR]** | 0.21 [0.13, 0.29] | 0.16 [0.10, 0.22] | 0.99 |
| **NK:** |  |  |  |
| **NK, median [IQR]** | 0.13 [0.09, 0.22] | 0.12 [0.08, 0.16] | 0.99 |
| **B cells:** |  |  |  |
| **Naive B, median [IQR]** | 0.41 [0.21, 0.50] | 0.74 [0.66, 0.84] | <0.001 |
| **USM B, median [IQR]** | 0.08 [0.05, 0.13] | 0.05 [0.03, 0.07] | <0.001 |
| **SM B, median [IQR]** | 0.26 [0.18, 0.34] | 0.10 [0.07, 0.14] | <0.001 |
| **DN B, median [IQR]** | 0.10 [0.06, 0.14] | 0.04 [0.03, 0.05] | <0.001 |
| **Plasmablast, median [IQR]** | 0.09 [0.04, 0.17] | 0.04 [0.02, 0.06] | <0.001 |
| **Monocytes:** |  |  |  |
| **CL Mono, median [IQR]** | 0.87 [0.84, 0.90] | 0.82 [0.77, 0.85] | <0.001 |
| **Int Mono, median [IQR]** | 0.03 [0.02, 0.05] | 0.04 [0.03, 0.05] | 0.99 |
| **NC Mono, median [IQR]** | 0.04 [0.02, 0.05] | 0.07 [0.05, 0.11] | <0.001 |
| **Dendritic cells (DC):** |  |  |  |
| **mDC, median [IQR]** | 0.01 [0.01, 0.01] | 0.01 [0.01, 0.02] | 0.12 |
| **pDC, median [IQR]** | 0.01 [0.00, 0.01] | 0.01 [0.01, 0.01] | 0.063 |

IQR = interquartile range

**Supplementary Table 6. Clinical features of the stratified patients with SLE**

|  | **Cluster 1 (n=28)** | **Cluster 2 (n=42)** | **p-value** |
| --- | --- | --- | --- |
| **Male sex (%)** | 4 (14.3) | 5 (11.9) | 0.99 |
| **Age, years, median [IQR]** | 47.50 [38.50, 54.25] | 47.00 [40.25, 58.75] | 0.742 |
| **Disease duration, years, median [IQR]** | 13.00 [5.00, 18.00] | 7.50 [0.50, 17.50] | 0.19 |
| **Acute cutaneous lupus (%)** | 17 (60.7) | 22 (52.4) | 0.624 |
| **Chronic cutaneous lupus (%)** | 6 (21.4) | 9 (21.4) | 0.99 |
| **Oral ulcers (%)** | 8 (28.6) | 11 (26.2) | 0.99 |
| **Nonscarring alopecia (%)** | 8 (28.6) | 16 (38.1) | 0.452 |
| **Synovitis (%)** | 21 (75.0) | 30 (71.4) | 0.79 |
| **Serositis (%)** | 6 (21.4) | 11 (26.2) | 0.779 |
| **Lupus nephritis (%)** | 11 (39.3) | 24 (57.1) | 0.222 |
| **Hemolytic anemia (%)** | 2 (7.1) | 8 (19.0) | 0.296 |
| **Leukopenia (%)** | 23 (82.1) | 35 (83.3) | 0.99 |
| **Thrombocytopenia (%)** | 8 (28.6) | 12 (28.6) | 0.99 |
| **Antiphospholipid syndrome (%)** | 5 (17.9) | 12 (29.3) | 0.395 |
| **Interstitial lung disease (%)** | 4 (14.3) | 6 (14.3) | 0.99 |
| **Serological findings** |  |  |  |
| **Antinuclear antibody (%)** | 28 (100) | 42(100) | 0.99 |
| **Anti-dsDNA antibody (%)** | 7 (25.0) | 18 (42.9) | 0.203 |
| **Anti-dsDNA antibody, IU/mL, median [IQR]** | 6.40 [0.95, 14.03] | 5.85 [1.30, 26.65] | 0.252 |
| **Anti-Sm antibody (%)** | 5/25 (20.0) | 8/39 (20.5) | 0.99 |
| **Anti-Sm antibody, IU/mL, median [IQR]** | 1.90 [0.90, 6.50] | 2.30 [1.70, 6.65] | 0.295 |
| **Anti-RNP antibody (%)** | 9/24 (37.5) | 11/38 (28.9) | 0.580 |
| **Anti-RNP antibody, IU/mL, median [IQR]** | 4.80 [0.70, 18.50] | 3.65 [1.83, 12.55] | 0.728 |
| **Anti-SS-A antibody (%)** | 15/19 (78.9) | 19/36 (52.8) | 0.082 |
| **Anti-SS-A antibody, IU/mL, median [IQR]** | 66.10 [12.30, 240.00] | 26.05 [1.37, 240.00] | 0.192 |
| **Anti-cardiolipin antibody (%)** | 4/21 (19.0) | 17/32 (53.1) | 0.021 |
| **Anti-cardiolipin antibody, IU/mL, median [IQR]** | 8.00 [8.00, 8.00] | 10.00 [8.00, 24.75] | 0.007 |
| **Anti-β2-glycoprotein-I antibody (%)** | 2/25 (8.0) | 11/38 (28.9) | 0.059 |
| **Lupus anticoagulant (%)** | 4/22 (18.2) | 14/38 (36.8) | 0.155 |
| **Lupus anticoagulant, median [IQR]** | 1.15 [1.10, 1.29] | 1.25 [1.16, 1.37] | 0.087 |
| **Hemoglobin, g/dL, median [IQR]** | 12.05 [11.28, 13.43] | 12.65 [11.03, 13.28] | 0.876 |
| **Hematocrit, %, median [IQR]** | 38.25 [34.70, 40.55] | 38.10 [33.30, 40.53] | 0.657 |
| **Platelet, 10³/μL, median [IQR]** | 20.75 [17.98, 26.15] | 21.80 [17.75, 25.70] | 0.742 |
| **Proteinuria, g/gCr, median [IQR]** | 0.08 [0.05, 0.24] | 0.12 [0.05, 0.32] | 0.414 |
| **Estimated glomerular filtration rate, mL/min/1.73m^2^, median [IQR]** | 76.25 [62.73, 89.97] | 75.55 [58.85, 87.05] | 0.900 |
| **50% Hemolytic Complement (U/mL), median [IQR]** | 49.25 [40.75, 54.45] | 42.00 [31.70, 52.30] | 0.152 |
| **C3,** **mg/dL, median [IQR]** | 72.50 [67.75, 92.50] | 75.00 [60.25, 94.25] | 0.867 |
| **C4, mg/dL, median [IQR]** | 16.50 [13.25, 21.25] | 15.00 [9.00, 20.00] | 0.217 |
| **C-reactive protein, mg/dL, median [IQR]** | 0.06 [0.03, 0.18] | 0.09 [0.03, 0.48] | 0.188 |
| **Disease Activity** |  |  |  |
| **SLE Disease Activity Index 2000, median [IQR]** | 2.00 [1.50, 4.50] | 4.50 [2.50, 8.00] | 0.042 |
| **SLICC/ACR Damage Index, median [IQR]** | 1.00 [0.00, 2.00] | 0.00 [0.00, 1.00] | 0.106 |
| **Physician Global Assessment [IQR]** | 1.00 [0.00, 3.00] | 3.00 [1.00, 6.00] | 0.006 |
| **Treatment** |  |  |  |
| **Prednisolone (%)** | 26 (92.9) | 32 (76.2) | 0.106 |
| **Hydroxychloroquine (%)** | 10 (35.7) | 15 (35.7) | 0.99 |
| **Mycophenolate mofetil (%)** | 3 (10.7) | 7 (16.7) | 0.729 |
| **Azathioprine (%)** | 9 (32.1) | 0 (0.0) | <0.001 |
| **Tacrolimus (%)** | 5 (17.9) | 4 (9.5) | 0.468 |
| **Cyclosporine A (%)** | 4 (14.3) | 3 (7.1) | 0.426 |
| **Methotrexate (%)** | 1 (3.6) | 2 (4.8) | 0.99 |

IQR = interquartile range

**Supplementary Table 7. Clinical features of the stratified patients with IIM.**

|  | **Cluster 1 (n=14)** | **Cluster 2 (n=40)** | **p-value** |
| --- | --- | --- | --- |
| **Male sex (%)** | 1 (7.1%) | 12 (30.0%) | 0.146 |
| **Age, years, median [IQR]** | 64.00 [56.25, 70.75] | 57.00 [51.25, 67.50] | 0.192 |
| **Disease duration, years, median [IQR]** | 8.00 [3.25, 10.75] | 2.50 [0.00, 14.25] | 0.209 |
| **Sample taken at disease onset (%)** | 3 (21.4) | 20 (50.0) | 0.115 |
| **Skin rash (%)** | 12 (85.7) | 29 (72.5) | 0.475 |
| **Heliotrope rash (%)** | 6 (42.9) | 11/39 (28.2) | 0.336 |
| **Gottron's sign (%)** | 11 (78.6) | 25/39 (64.1) | 0.506 |
| **Mechanic's hands (%)** | 4 (28.6) | 14/39 (35.9) | 0.748 |
| **Interstitial lung disease (%)** | 8 (57.1) | 23 (57.5) | 0.99 |
| **Coexistence of malignancy (%)** | 0 (0.0) | 1 (2.5) | 0.99 |
| **Clinical Diagnoses** |  |  |  |
| **Dermatomyositis (%)** | 8 (57.1) | 20 (50.0) | 0.76 |
| **Clinically amyopathic dermatomyositis (%)** | 4 (28.6) | 8 (20.0) | 0.485 |
| **Polymyositis (%)** | 2 (14.3) | 6 (15.0) | 0.99 |
| **Immune-mediated necrotizing myopathy (%)** | 0 (0.0) | 3 (7.5) | 0.56 |
| **Inclusion body myositis (%)** | 0 (0.0) | 2 (5.0) | 0.99 |
| **Autoantibodies** |  |  |  |
| **Anti-synthetase antibody (%)** | 4 (28.6) | 12 (30.0) | 0.99 |
| **Anti-melanoma differentiation-associated gene 5 antibody (%)** | 2 (14.3) | 9 (22.5) | 0.708 |
| **Anti-Mi-2 antibody (%)** | 2 (14.3) | 5 (12.5) | 0.99 |
| **Anti-transcription intermediary factor 1-gamma antibody (%)** | 1 (7.1) | 1 (2.5) | 0.455 |
| **Anti-signal recognition particle antibody (%)** | 0 (0.0) | 2 (6.1) | 0.99 |
| **Anti-3-hydroxy-3-methylglutaryl-CoA reductase antibody (%)** | 0 (0.0) | 1 (3.1) | 0.99 |
| **Anti-SS-A antibody (%)** | 2 (14.3) | 11 (27.5) | 0.475 |
| **Myositis-specific antibody negative (%)** | 5/14 (35.7) | 8 (20.0) | 0.285 |
| **Other Findings** |  |  |  |
| **AST, U/L, median [IQR]** | 26.00 [20.00, 48.00] | 27.50 [19.00, 43.00] | 0.948 |
| **ALT, U/L, median [IQR]** | 17.00 [14.00, 55.00] | 25.50 [12.75, 37.50] | 0.967 |
| **Creatine kinase, U/L, median [IQR]** | 89.50 [62.75, 144.50] | 130.00 [74.00, 888.00] | 0.118 |
| **Peak creatine kinase, U/L, median [IQR]** | 315.00 [92.00, 1689.00] | 1094.50 [233.75, 2601.50] | 0.172 |
| **C-reactive protein, mg/dL, median [IQR]** | 0.05 [0.04, 0.10] | 0.11 [0.04, 0.36] | 0.242 |
| **Muscle biopsy** |  |  |  |
| **Perifascicular atrophy (%)** | 2/11 (18.2) | 2/35 (5.7) | 0.238 |
| **CD8^+^ T cell infiltration with MHC class I upregulation (%)** | 0/11 (0.0) | 2/34 (5.9) | 0.99 |
| **Treatment** |  |  |  |
| **Prednisolone (%)** | 12 (85.7) | 17 (42.5) | 0.006 |
| **Tacrolimus (%)** | 6 (42.9) | 5 (12.5) | 0.024 |
| **Azathioprine (%)** | 1 (7.1) | 0 (0.0) | 0.259 |
| **Cyclosporine A (%)** | 2 (14.3) | 2 (5.0) | 0.274 |
| **Methotrexate (%)** | 4 (28.6) | 5 (12.5) | 0.216 |

IQR = interquartile range

**Supplementary Table 8. Clinical features of the stratified patients with SSc.**

|  | **Cluster 1 (n=6)** | **Cluster 2 (n=46)** | **p-value** |
| --- | --- | --- | --- |
| **Male sex (%)** | 0 (0.0) | 3 (6.5) | 0.99 |
| **Age, years, median [IQR]** | 70.50 [65.50, 71.00] | 66.50 [55.25, 73.75] | 0.24 |
| **Disease duration, years, median [IQR]** | 18.50 [17.25, 22.00] | 15.00 [4.75, 27.50] | 0.431 |
| **Clinical Findings** |  |  |  |
| **Mean modified Rodnan skin score, median [IQR]** | 3.50 [1.62, 6.50] | 4.00 [2.00, 7.00] | 0.604 |
| **Arthralgia (%)** | 2 (33.3) | 23 (50.0) | 0.67 |
| **Gastroesophageal reflux disease (%)** | 5 (83.3) | 36 (78.3) | 0.99 |
| **Constipation or diarrhea (%)** | 3 (50.0) | 20 (43.5) | 0.99 |
| **Renal crisis (%)** | 1 (16.7) | 0 (0.0) | 0.115 |
| **Chronic kidney disease (%)** | 2 (33.3) | 3 (6.5) | 0.096 |
| **Shortness of breath** | 4 (66.7%) | 16 (34.8%) | 0.19 |
| **Interstitial lung disease (%)** | 5 (83.3) | 18 (39.1) | 0.11 |
| **% Vital capacity, median [IQR]** | 72.00 [58.25, 90.25] | 98.50 [80.30, 111.00] | 0.027 |
| **% Diffusing capacity for carbon monoxide, median [IQR]** | 73.50 [69.93, 77.00] | 86.50 [70.72, 100.75] | 0.071 |
| **Krebs von den Lungen-6, U/mL median [IQR]** | 497.50 [383.75, 560.25] | 319.00 [207.00, 501.00] | 0.339 |
| **C-reactive protein, mg/dL, median [IQR]** | 0.13 [0.06, 0.22] | 0.07 [0.02, 0.12] | 0.36 |
| **Autoantibodies** |  |  |  |
| **Anti-topoisomerase antibody (%)** | 2 (33.3) | 12 (26.1) | 0.655 |
| **Anti-centromere antibody (%)** | 1 (16.7) | 31 (67.4) | 0.026 |
| **Anti-RNA polymerase III antibody (%)** | 1 (16.7) | 2 (4.3) | 0.313 |
| **Treatment** |  |  |  |
| **Tacrolimus (%)** | 1 (16.7) | 3 (6.5) | 0.397 |
| **Mycophenolate mofetil (%)** | 1 (16.7) | 2 (4.3) | 0.313 |
| **Cyclosporine A (%)** | 1 (16.7) | 0 (0.0) | 0.115 |
| **Methotrexate (%)** | 0 (0.0) | 4 (8.7) | 0.99 |
| **Tocilizumab (%)** | 1 (16.7) | 3 (6.5) | 0.397 |

IQR = interquartile range

**Supplementary Table 9. Clinical features of the stratified patients with MCTD.**

|  | **Cluster 1 (n=4)** | **Cluster 2 (n=16)** | **p-value** |
| --- | --- | --- | --- |
| **Male sex (%)** | 1 (25.0) | 3 (18.8) | 0.99 |
| **Age, years, median [IQR]** | 56.50 [51.00, 64.25] | 50.00 [41.25, 67.00] | 0.321 |
| **Disease duration, years, median [IQR]** | 21.50 [19.25, 23.25] | 15.50 [8.75, 20.50] | 0.184 |
| **General Features** |  |  |  |
| **Raynaud's phenomenon (%)** | 4 (100.0) | 16 (100.0) |  |
| **Puffy fingers (%)** | 3 (75.0) | 16 (100.0) | 0.2 |
| **Aseptic meningitis (%)** | 1 (25.0) | 1 (6.2) | 0.368 |
| **Anti-U1-RNP antibody, IU/mL, median [IQR]** | 176.3 [136.4, 187.33] | 143.3 [109.1, 200] | 0.9096 |
| **Systemic lupus erythematosus Features** |  |  |  |
| **Arthritis (%)** | 2 (50.0) | 9 (56.2) | 0.99 |
| **Lymph node swelling (%)** | 1 (25.0) | 4 (25.0) | 0.99 |
| **Facial erythema (%)** | 1 (25.0) | 4 (25.0) | 0.99 |
| **Serositis (%)** | 2 (50.0) | 1 (6.2) | 0.088 |
| **Leukopenia (%)** | 0 (0.0) | 3 (18.8) | 0.99 |
| **Thrombocytopenia (%)** | 1 (25.0) | 2 (12.5) | 0.509 |
| **Systemic sclerosis features** |  |  |  |
| **Sclerodactyly (%)** | 1 (33.3) | 8 (50.0) | 0.99 |
| **Krebs von den Lungen-6, median [IQR]** | 431.00 [348.00, 667.00] | 345.00 [196.00, 482.00] | 0.459 |
| **Vital capacity, median [IQR]** | 82.55 [77.70, 90.00] | 90.65 [79.67, 1.18] | 0.595 |
| **Gastroesophageal reflux disease (%)** | 3 (75.0) | 7 (43.8) | 0.582 |
| **Myositis features** |  |  |  |
| **Muscle weakness of limbs (%)** | 0 (0.0) | 1 (6.2) | 0.99 |
| **Myalgia (%)** | 1 (25.0) | 1 (6.2) | 0.368 |
| **Elevated creatine kinase or aldolase (%)** | 0 (0.0) | 3 (18.8) | 0.99 |

IQR = interquartile range

**Supplementary Table 10. Clinical features of the stratified patients with RA.**

|  | **Cluster 1 (n=4)** | **Cluster 2 (n=16)** | **p-value** |
| --- | --- | --- | --- |
| **Male sex (%)** | 0 (0.0) | 0 (0.0) | 0.99 |
| **Age, years, median [IQR]** | 48.50 [41.25, 59.00] | 66.50 [59.75, 71.50] | 0.185 |
| **Disease duration, years, median [IQR]** | 26.00 [19.55, 26.00] | 8.90 [0.95, 20.50] | 0.201 |
| **Disease Activity** |  |  |  |
| **Clinical disease activity index, median [IQR]** | 32.45 [27.67, 36.25] | 13.00 [5.50, 23.50] | 0.02 |
| **Simplified disease activity index, median [IQR]** | 31.39 [25.97, 36.53] | 14.00 [7.12, 25.66] | 0.049 |
| **Health assessment questionnaire, median [IQR]** | 1.52 [1.39, 1.66] | 1.25 [0.75, 1.25] | 0.223 |
| **Interstitial lung disease (%)** | 0 (0.0) | 0 (0.0) |  |
| **Serological findings** |  |  |  |
| **C-reactive protein, mg/dL, median [IQR]** | 0.56 [0.14, 1.30] | 1.73 [0.84, 4.22] | 0.147 |
| **Erythrocyte sedimentation rate, mm/hr, median [IQR]** | 28.0 [13.5, 46.25] | 51.0 [23.75, 74.25] | 0.19 |
| **Rheumatoid factor, median [IQR]** | 18.50 [11.40, 119.50] | 27.00 [9.00, 67.00] | 0.571 |
| **Anti-cyclic citrullinated peptide antibody, IU/dL, median [IQR]** | 29.90 [0.60, 147.40] | 100.00 [21.05, 286.00] | 0.271 |
| **Treatment** |  |  |  |
| **Prednisolone, mg/day, median [IQR]** | 3.25 [0.00, 7.38] | 0.00 [0.00, 2.62] | 0.285 |
| **Methotrexate, mg/week, median [IQR]** | 1.00 [0.00, 5.50] | 0.00 [0.00, 8.50] | 0.669 |
| **Tacrolimus (%)** | 0 (0.0) | 1 (6.2) | 0.99 |
| **Iguratimod (%)** | 1 (25.0) | 3 (18.8) | 0.99 |

IQR = interquartile range

**Supplementary Table 11. Clinical features of the stratified patients with LVV.**

|  | **Cluster 1 (n=10)** | **Cluster 2 (n=9)** | **p-value** |
| --- | --- | --- | --- |
| **Age, years, median [IQR]** | 72.00 [60.50, 76.75] | 44.00 [33.00, 67.00] | 0.05 |
| **Male sex (%)** | 1 (10.0%) | 2 (22.2%) | 0.582 |
| **Disease duration, years, median [IQR]** | 30.00 [22.25, 38.75] | 9.00 [1.00, 20.00] | 0.016 |
| **Diagnosis and serological findings** |  |  |  |
| **Takayasu arteritis (%)** | 9 (90.0) | 7 (77.8) | 0.582 |
| **Giant cell arteritis (%)** | 1 (10.0) | 2 (22.2) | 0.582 |
| **Coexistence of polymyalgia rheumatica (%)** | 1 (10.0) | 2 (22.2) | 0.582 |
| **C-reactive protein, mg/dL, median [IQR])** | 0.08 [0.04, 0.16] | 0.21 [0.16, 0.30] | 0.093 |
| **Treatment** |  |  |  |
| **Prednisolone, mg/day, median [IQR]** | 5.00 [4.13, 6.50] | 5.00 [3.00, 7.50] | 0.7941 |
| **Azathioprine (%)** | 2 (20.0) | 1 (11.1) | 0.99 |
| **Cyclosporine A (%)** | 1 (10.0) | 0 (0.0) | 0.99 |
| **Methotrexate (%)** | 3 (30.0) | 1 (11.1) | 0.582 |
| **Tacrolimus (%)** | 0 (0.0) | 2 (22.2) | 0.211 |

IQR = interquartile range

**Supplementary Table 12. Clinical features of the stratified patients with LVV.**

|  | Types of flare |
| --- | --- |
| SLE | Arthritis, n=8; lupus nephritis (urinary casts or proteinuria), n=5; thrombocytopenia, n=4; low complement, n=3; leukopenia, n=2; alopecia, n=2; fever, n=2; rash, n=1; vasculitis, n=1: thrombotic microangiopathy, n=1; interstitial lung disease, n=1 |
| IIM | Interstitial lung disease, n=2; skin rash, n=1; myositis n=1 |
| SSc | Interstitial lung disease, n=7; arthritis, n=5; digital tip ulcers, n=2: skin thickening of the fingers, n=1; myositis, n=1 |
| MCTD | Arthritis, n=2; interstitial lung disease, n=2; leukopenia, n=1; puffy finger, n=1; low complement, n=1: pleurisy, n=1 |
| RA | Arthritis, n=7 |
| LVV | Vasculitis, n=3 |

**Supplementary Table 13.** **Associations of age-associated B cell signature scores in B-cell subsets with SLEDAI-2K and SDI.**

|  | Multiple linear regression model | | | | Pearson correlation analyses | |
| --- | --- | --- | --- | --- | --- | --- |
|  | Regression coefficient (β) | Standard Error | t-statistic | Adjusted  p-value | Correlation Coefficient (r) | Adjusted  p-value |
| SLEDAI-2K |  |  |  |  |  |  |
| Naive B | -2.52 | 2.27 | -1.11 | 0.35 | -0.086 | 0.60 |
| USM B | -1.41 | 1.28 | -1.10 | 0.35 | -0.068 | 0.60 |
| SM B | 1.90 | 1.57 | 1.21 | 0.35 | 0.23 | 0.35 |
| DN B | 0.66 | 1.34 | 0.49 | 0.63 | 0.17 | 0.35 |
| Plasmablast | 1.58 | 1.29 | 1.23 | 0.35 | 0.16 | 0.35 |
| SDI |  |  |  |  |  |  |
| Naive B | -0.87 | 0.39 | -2.21 | 0.16 | -0.31 | 0.079 |
| USM B | 0.20 | 0.25 | 0.81 | 0.70 | -0.0058 | 0.96 |
| SM B | -0.25 | 0.27 | -0.92 | 0.70 | -0.25 | 0.14 |
| DN B | 0.0023 | 0.24 | 0.0097 | 0.99 | -0.16 | 0.38 |
| Plasmablast | 0.098 | 0.23 | 0.42 | 0.85 | 0.026 | 0.96 |

Multiple linear regression and Pearson correlation analyses examining the relationship between ABC signature scores and the SLEDAI-2K and the SDI. The regression model included age, sex, and disease duration as covariates. P-values were adjusted using the Benjamini–Hochberg method.

ABC = age-asscoaited B cell**,** SLEDAI-2K = SLE Disease Activity Index 2000, SDI = Systemic Lupus International Collaborating Clinics/American College of Rheumatology Damage Index.
